# Genome specialization and decay of the strangles pathogen, *Streptococcus equi*, is driven by persistent infection

**DOI:** 10.1101/gr.189803.115

**Published:** 2015-09

**Authors:** Simon R. Harris, Carl Robinson, Karen F. Steward, Katy S. Webb, Romain Paillot, Julian Parkhill, Matthew T.G. Holden, Andrew S. Waller

**Affiliations:** 1 The Wellcome Trust Sanger Institute, Wellcome Trust Genome Campus, Hinxton, Cambridge CB10 1SA, United Kingdom;; 2 The Animal Health Trust, Lanwades Park, Kentford, Newmarket CB8 7UU, United Kingdom;; 3 School of Medicine, University of St Andrews, North Haugh, St. Andrews KY16 9TF, United Kingdom

## Abstract

Strangles, the most frequently diagnosed infectious disease of horses worldwide, is caused by *Streptococcus equi*. Despite its prevalence, the global diversity and mechanisms underlying the evolution of *S. equi* as a host-restricted pathogen remain poorly understood. Here, we define the global population structure of this important pathogen and reveal a population replacement in the late 19th or early 20th Century. Our data reveal a dynamic genome that continues to mutate and decay, but also to amplify and acquire genes despite the organism having lost its natural competence and become host-restricted. The lifestyle of *S. equi* within the horse is defined by short-term acute disease, strangles, followed by long-term infection. Population analysis reveals evidence of convergent evolution in isolates from post-acute disease samples as a result of niche adaptation to persistent infection within a host. Mutations that lead to metabolic streamlining and the loss of virulence determinants are more frequently found in persistent isolates, suggesting that the pathogenic potential of *S. equi* reduces as a consequence of long-term residency within the horse post-acute disease. An example of this is the deletion of the equibactin siderophore locus that is associated with iron acquisition, which occurs exclusively in persistent isolates, and renders *S. equi* significantly less able to cause acute disease in the natural host. We identify several loci that may similarly be required for the full virulence of *S. equi*, directing future research toward the development of new vaccines against this host-restricted pathogen.

Bacterial pathogens live a double life, balancing the requirements of acute disease with persistence and colonization in order to optimize their ability to transmit to naïve animals. These high and low virulence states evolve over time to fit with each host species. However, the impact of evolution toward a persistent state on streamlining the genome of a virulent organism has not been described. Strangles is one of the oldest recognized infectious diseases of horses and continues to cause significant welfare and economic costs throughout the world. The causative agent, *Streptococcus equi* subspecies *equi* (*S. equi*), was first identified in 1888 (Schutz 1888). However, the clinical signs of strangles, typified by pyrexia, followed by abscessation of lymph nodes in the head and neck, were first reported by Jordanus Ruffus in 1251 (Ruffus 1251), and detailed descriptions of the clinical course of disease are provided in old veterinary texts (Solleysel 1664; White 1825; Anon 1831; Blaine 1841; White and Spooner 1842; Miles 1868; Williams 1884). The historical clinical signs of strangles exactly mimic those observed in contemporary populations of horses, and it is likely that the disease has been caused by the same species of bacteria throughout this 700-yr period. Rupture of lymph node abscesses releases highly infectious pus that can spread the infection from one horse to another, but this direct transmission from acute disease cases in itself cannot explain the global success of *S. equi*. Rather, it is believed that incomplete drainage of abscess material from the retropharyngeal lymph nodes permits the organism to persistently infect the adjacent guttural pouches of convalescent horses, usually by residing within dried balls of pus known as chondroids (Newton et al. 1997). *S. equi* can persist in this low nutrient state in the absence of clinical signs for several years and potentially the remaining lifetime of the horse, providing the organism with prolonged opportunity to be shed into the environment and transmit to naïve animals (Newton et al. 2000). The disease cycle of acute disease followed by persistent infection has underpinned the success of *S. equi*, balancing the requirements of both acute and persistent states.

The worldwide population of *S. equi* is almost clonal by multilocus sequence typing (MLST) (Webb et al. 2008), consisting of only two sequence types (ST), ST179 and ST151, and has been proposed to have evolved recently from an ancestral strain of *Streptococcus equi* subspecies *zooepidemicus* (*S. zooepidemicus*) (Webb et al. 2008). Both *S. equi* and *S. zooepidemicus* belong to the same group of pyogenic streptococci as the important human pathogen *Streptococcus pyogenes* (Group A *Streptococcus*) and share many common virulence mechanisms, with evidence of cross-species horizontal DNA exchange (Holden et al. 2009; Lefebure et al. 2012). Comparison of the complete genomes of *S. equi* strain 4047 (*Se*4047) and *S. zooepidemicus* strain H70 (*Sz*H70) showed that the pathogenic specialization of *S. equi* has been shaped by a combination of gene loss due to nonsense mutations and deletions, and gene gain through the acquisition of mobile genetic elements (MGEs) (Holden et al. 2009). However, the effects of the disease cycle transitions between acute disease and persistent infection on the genome of *S. equi* remain unknown.

In this study, we utilize whole-genome sequencing of a large collection of *S. equi* strains from around the globe. The collection contains isolates from horses that were part of outbreaks on large farms in the United Kingdom, including isolates from acute and persistent states and also multiple isolates from the same animal. Using these data, we investigate the evolutionary history of the species, reveal the complexity of disease outbreaks and uncover evidence of common pathways of within-host adaptation associated with persistent infection, which together shed new light on the evolution of this important animal pathogen toward host restriction.

## Results

### Population structure of a global collection of *S. equi*

The genomes of a collection of 224 *S. equi* isolates were sequenced (Supplemental Table 1). Forty geographically and temporally diverse isolates from outbreaks in Australia, Belgium, Canada, Ireland, New Zealand, Saudi Arabia, Sweden, and the United States were included to provide a global snapshot of the diversity of *S. equi*. Allied with this, 180 acute infection and persistent isolates from 41 counties across the United kingdom were included to determine the diversity of *S. equi* within individual outbreaks. Finally, isolates from commercially available live attenuated vaccines, Equilis StrepE (Jacobs et al. 2000) and Pinnacle IN (high and low capsule) (Walker and Timoney 2002), and the *Se*1866 isolate, which is the basis of the new multicomponent Strangvac vaccine (Guss et al. 2009), were included to determine the relationships of these vaccines to the currently circulating population of *S. equi* and to identify mutations responsible for attenuation of the Pinnacle IN strain.

The indexed sequencing reads from each isolate were mapped against the *Se*4047 reference genome (Holden et al. 2009) to identify single-nucleotide polymorphisms (SNPs), insertions, and deletions (indels). Across the genome, 3109 polymorphic sites were identified. Of these sites, 58.8% (*n* = 1844) were in accessory regions of the *Se*4047 reference genome, which corresponds to only 16.4% of the length of the genome. Much of this variation in accessory regions is likely to have arisen by recombination and horizontal gene transfer of alternative MGEs. To remove confounding signals in these regions, all phylogenetic analyses were carried out using the 1265 polymorphic sites (Supplemental Table 2) in the 1.9 Mb of the core genome.

**Figure 1.**
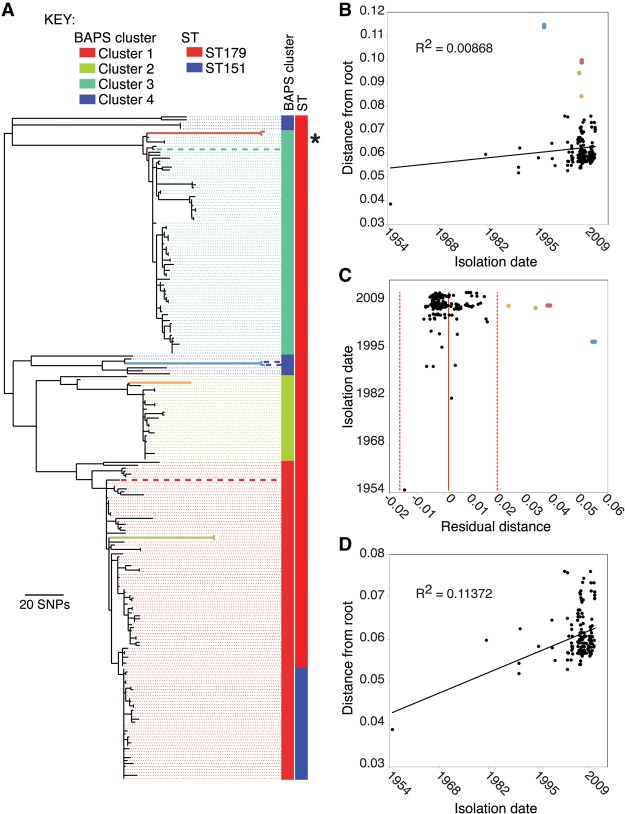
Phylogenetic reconstruction and assessment of temporal signal. (*A*) Maximum-likelihood phylogeny of *S. equi* based on core genome SNPs. Clusters are colored as reconstructed by BAPS. Columns adjacent to the tree represent the BAPS cluster and MLST type associated with the strains. Dashed lines linking branches to these columns are for ease of viewing and are colored with the appropriate BAPS cluster color. Branch colors represent branches corresponding to isolates from horses *JKS628* (red), *JKS731* (orange), *JKS121* (green), and two Pinnacle vaccine strains (blue). Thick dashed lines indicate vaccine strains: (blue) Pinnacle IN; (green) Equilis Strep E; and (red) *Se*1866, the basis of the Strangvac vaccine. Note that the dashed blue lines for the Pinnacle IN vaccine isolates are short relative to the long branches for these isolates, which are highlighted in blue by virtue of their raised substitution rates. The position of the reference isolate, *Se*4047, is indicated by an asterisk. (*B*) Root-to-tip plot of all isolates in the ML tree in *A*. (*C*) Residual plot of root-to-tip plot in *B*. Solid red line indicates the mean, and dashed red lines indicate two standard deviations from the mean. (*D*) Root-to-tip plot with the isolates falling outside of the 95% confidence intervals in *B* excluded. Colored points in *B* and *C* correspond to the isolates on colored branches in *A*.

A maximum likelihood (ML) phylogenetic reconstruction of the core variable sites of the sequenced isolates is shown in Figure 1A. Isolates are colored according to clusters defined using a tree-independent Bayesian method for subdividing populations based on sequence similarity (Corander et al. 2008). Clusters 1–3 correspond to the three major clades in the ML tree, with cluster 4 representing the remaining isolates. The increasingly prevalent ST151 formed a clade within cluster 1, distant from the Equilis StrepE and Pinnacle IN vaccine samples, which fell into clusters 3 and 4, respectively (Fig. 1A). Two features of the tree were particularly striking. First, the observed sequence diversity was surprisingly low for a global collection of isolates of a pathogen with a historical record that suggests it dates back to at least the 13th Century. Second, the tree includes a number of anomalously long branches toward its tips, which appeared inconsistent with the isolation dates of the samples.

### Variation in the rate of substitution in the *S. equi* core genome

Plotting root-to-tip distance against isolation date for each isolate for which dating information was available revealed a poor correlation between accumulation of substitutions and time (Fig. 1B) (*R*^2^ = 0.00868), although a tip-date permutation test (Firth et al. 2010) confirmed significant temporal signature in the data (*P* = 0.001 from 1000 permutations). Similar observations in other streptococci have been the result of the effect of homologous recombination, which can import large amounts of variation into the genome en masse, leading to elongation of branches and the loss of clock-like mutational signals. Although it has been thought that *S. equi* has lost natural competence (Holden et al. 2009), we investigated the possibility that recombination was the cause of the observed long branches. By reconstructing sequences for ancestral nodes in the tree, evidence for past recombination events can be identified in two ways: Recombination events from donors outside the diversity of the tree will cause imports of large numbers of clustered substitutions on individual branches of the tree, whereas recombination events between donors and recipients within the diversity of the tree will lead to accumulation of homoplasies. In contrast to other streptococci (Croucher et al. 2011; Marttinen et al. 2011; Cornejo et al. 2013), we observed low levels of both SNP clustering (Supplemental Fig. 1) and homoplasy (only 47 or 3.6% of core variable sites), ruling out recombination as the cause of the long branches. The highest density of SNPs (3.2% of SNPs, *P* < 0.0001) and homoplasies (28% of homoplasic SNPs, *P* < 0.0001) in the core genome coincided with the 5′ 1.2-kb region (0.06% of the core genome length) of the SeM protein gene (*seM*) that encodes a virulence factor that binds to fibrinogen and immunoglobulin. The promoter region of the same gene also contained three homoplasies. Similarly, genes encoding the fibronectin- or fibrinogen-binding proteins, FneE and SzPSe, also exhibit high SNP density and homoplasy, highlighting the importance to *S. equi* of generating variation in these proteins.

Given the observed lack of homologous recombination, the poor root-to-tip correlation must have resulted from variation in substitution rate across the tree. Plotting the residuals for the root-to-tip analysis (Fig. 1C) showed that seven isolates, subtended by four long branches when mapped onto the tree (Fig. 1A), fell outside of two standard deviations of the mean. These included two isolates of the Pinnacle IN vaccine strain; two isolates extracted from the right guttural pouch of a horse (*JKS121;* italicized text indicates the ID of the horse; standard text indicates the *S. equi* isolate from this animal, in this case isolates, JKS121a and JKS121b) sampled during a strangles outbreak in Leicestershire; two isolates from the guttural pouches of a horse (*JKS628*) sampled during an outbreak in Essex; and one isolate from the right guttural pouch of a horse (*JKS731*) sampled during an outbreak in Lincolnshire. Excluding these seven isolates increased the root-to-tip *R*^2^ value to 0.11372 (Fig. 1D). The long branch associated with the Pinnacle IN isolates resulted from 68 shared unique SNPs in the core genome of these isolates. This diversity could be explained by the methods used to make the vaccine, which was a live attenuated vaccine generated by chemical mutagenesis of *S. equi* with *N*-methyl-*N*′-nitro-*N*-nitrosoguanidine (NTG) (Timoney 1985). As expected for NTG-treatment, the substitution spectra for the SNPs unique to the Pinnacle isolates was significantly enriched for C→T and G→A, but deficient in A→G and T→C transitions (Harper and Lee 2012) when compared to other branches in the tree (Supplemental Fig. 2). In wild-type isolates, disruption of the mismatch repair system can also lead to increased substitution rates, but is characterized by an increase in the accumulation of A→G and T→C transitions. We found no significant differences between the substitution spectra of the three long branches leading to clinical isolates, including two isolates from JKS121 that exhibited a nonsynonymous mutation in the mismatch repair gene, *mutS* (Supplemental Fig. 2). Consistent with this, no significant differences (*P* = 0.093) in resistance mutation frequency were found when comparing the long-branch isolates from *JKS121*, *JKS628*, *JKS731*, the Pinnacle IN vaccine isolates, and the reference *Se*4047 in vitro (Supplemental Fig. 3).

The two isolates grown from the Pinnacle IN vaccine differed phenotypically in their amount of hyaluronic acid capsule (Walker and Timoney 2002), hemolysis of red blood cells, and by three SNPs, including a nonsynonymous substitution (Ile206Thr) in a homolog of the *csrS* two-component system sensor that has been demonstrated to regulate virulence factors, including capsule, in *S. pyogenes* (Graham et al. 2002). Both isolates shared a second SNP in *csrS*, which is predicted to result in a Leu257Phe amino acid substitution in addition to one nonsense mutation and a further 40 nonsynonymous mutations elsewhere in the genome (Supplemental Table 2). The combination of these SNPs likely explain the attenuation of Pinnacle and could now form the basis of a diagnostic test to resolve cases when the vaccine strain has been suggested to have caused adverse reactions or reverted to virulence (Borst et al. 2011; Patty and Cursons 2014).

### Sequencing resolved the complex epidemiology of strangles outbreaks

**Figure 2.**
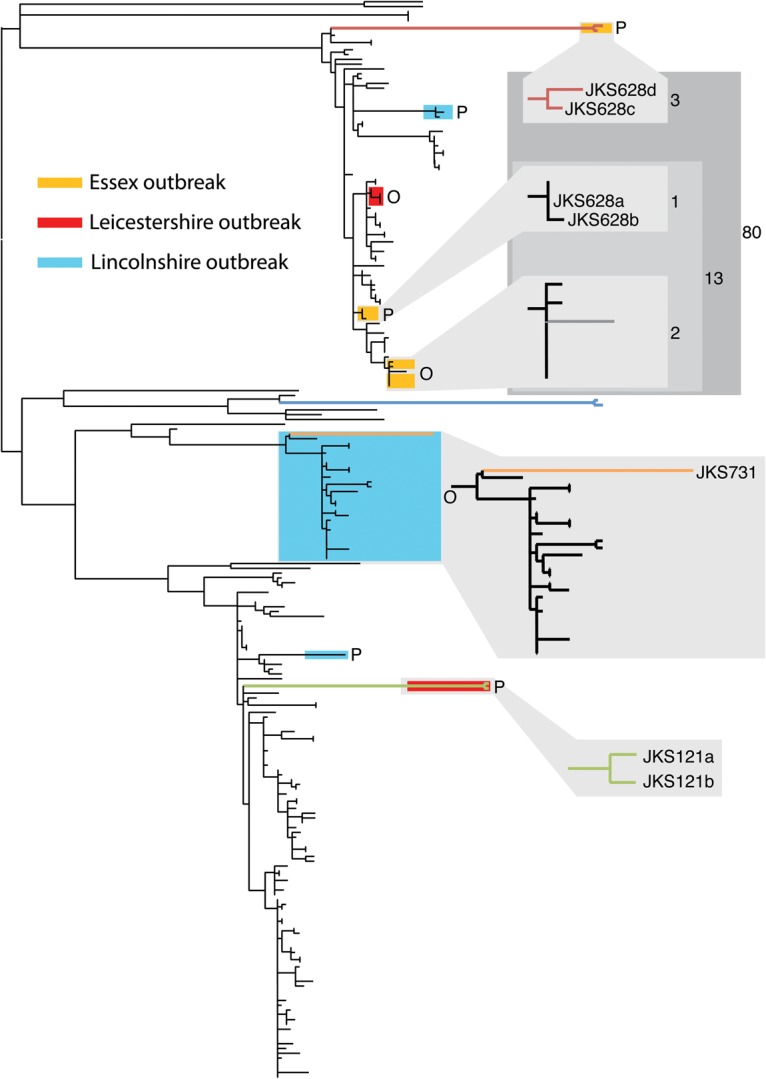
Detailed epidemiology of three *S. equi* outbreaks in the United Kingdom. Isolates highlighted on the phylogeny were collected from three stables experiencing strangles outbreaks, as indicated by the key. In each case, isolates did not fall into single clades on the phylogeny; instead they were grouped into outbreak (O) and persistent (P) clusters. The positions of the outlier branches identified by root-to-tip analysis are labeled in blowups of the relevant clades. For the Essex outbreak, the number of SNPs within and between clades is indicated by the numbers in the gray boxes. Colored branches in the tree correspond to those in Figure 1.

*JKS121*, *JKS628*, and *JKS731* were all sampled during strangles outbreaks in the United Kingdom. The inclusion of multiple isolates from outbreaks allows us to utilize the resolution that whole-genome sequencing provides to conduct detailed epidemiological analysis of strangles. In most cases, outbreak isolates were highly clonal and differed by only a small number of SNPs (Supplemental Fig. 4), consistent with an import from a single source. However, in some cases, including the outbreaks involving *JKS121*, *JKS628*, and *JKS731*, both active strangles strains and persistent strains from chondroids were identified from horses at the same stables during a strangles outbreak (Supplemental Fig. 4). In one case, 10 isolates recovered from a small outbreak in Essex over a 5-mo period all fell into cluster 3 on our phylogeny. However, within this cluster, the isolates grouped into three distinct subclades differentiating an outbreak strain and two persistent strains (Fig. 2). Interestingly, of the four isolates recovered from *JKS628*, including the two outliers in our root-to-tip analysis, two fell into each of the persistent clades, possibly reflecting separate, long-standing persistent infections prior to the purchase of this horse some 15 mo before the outbreak. A similar situation was observed for six isolates from an outbreak in Leicestershire in 2007. Four isolates, from both persistent infection and disease, formed a clade in cluster 3, representing the outbreak strain. However, the two root-to-tip outliers from *JKS121* were distinct, falling into cluster 1. These two persistent isolates possibly originated from a previous outbreak during the 8-yr residency of this horse on the affected premises. Finally, *JKS731* was sampled during a large outbreak in Lincolnshire that persisted from 2006 to 2008 and affected more than 200 horses. Twenty-seven isolates from this outbreak formed a single subclade within cluster 2 on our tree. Two of these isolates, including the persistent isolate from *JKS731*, which was the final outlier in our root-to-tip analysis, shared a much deeper common ancestor than the rest of the outbreak isolates, which suggests these may be persistent isolates from a historic outbreak of a similar genotype, possibly occurring during the 15-yr residency of this horse at the affected premises. The outbreak strain may have been the result of reinfection from an external source, or more likely from a persistent infection at the stables. As with the Essex and Leicestershire outbreaks, isolates taken during the Lincolnshire outbreak also included two persistent lineages that clustered far away from the main outbreak clade, illustrating the prevalence of long-term persistence of *S. equi*.

### Substitution rates vary between acute infection and persistence

**Figure 3.**
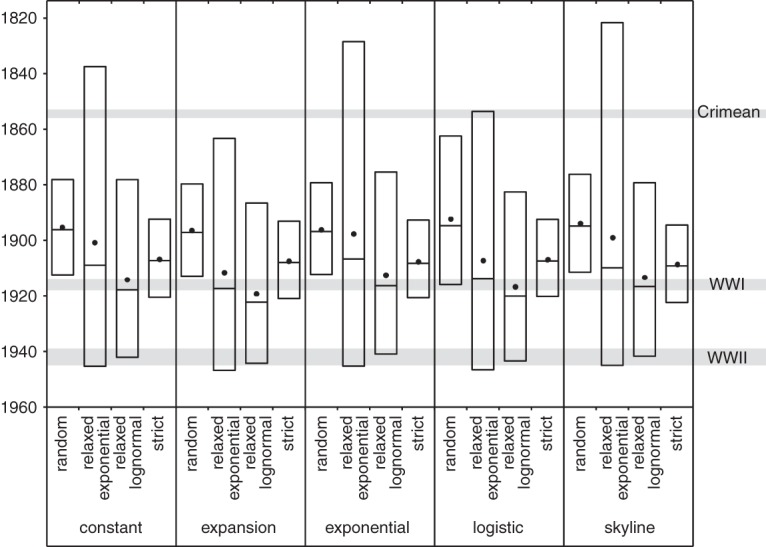
Box plots showing mean (dots), median (*center* horizontal line), and 95% HPD (highest posterior density) for the time to the most recent common ancestor (tMRCA) of all isolates in the study under various combinations of clock and population model in BEAST. The dates of the Crimean War, World War I (WWI), and World War II (WWII) are indicated by the gray shaded regions.

Remarkably, despite the five clinical isolates identified as outliers by our root-to-tip analysis being sampled during outbreaks, all were isolated from chondroids extracted from the guttural pouches of healthy horses, and therefore were representative of persistent populations that originated from historic acute infections. This observation raised the possibility that the poor temporal signal identified in our root-to-tip analysis was the result of *S. equi* displaying altered substitution rates during persistent infection. To shed further light on this, we analyzed the subset of our data set for which accurate isolation dates were available with the Bayesian phylogenetics software, BEAST (Drummond and Rambaut 2007), which allows modeling of molecular clock rates to provide estimations of divergence dates on nodes of a phylogenetic tree. Removal of artificially attenuated vaccine isolates and isolates without dating information produced a data set of 209 isolates with 1184 polymorphic core genome positions. BEAST includes a number of relaxed molecular clock models that permit modeling of variation in substitution rates on different branches of the tree, allowing us to correct for the observed rate variation in our data and also to identify other branches exhibiting particularly high substitution rates. Indeed, the combination of a skyline population model and relaxed-exponential clock model was found to be the best fit to our data based on Bayes factors using the harmonic mean estimator. The topology of the maximum clade credibility (MCC) tree generated from combined data post-burn-in from three independent runs of this model was concordant with the ML tree of all isolates (Supplemental Fig. 5). The mean substitution rate per core genome site per year was calculated as 5.22 × 10^−7^ (95% highest posterior density [HPD]: 4.04 × 10^−7^ to 6.51 × 10^−7^), slower than the core genome rates reported for other streptococci, including *S. pyogenes* (1.1 × 10^−6^) (Davies et al. 2015), *Streptococcus pneumoniae* (1.57 × 10^−6^) (Croucher et al. 2011), and many other gram-positive bacteria, including *Staphylococcus aureus* (3.3 × 10^−6^) (Harris et al. 2010; Holden et al. 2013). Interestingly, the mutation rate of *S. equi* is closer to those calculated for *Clostridium difficile* (1.47 × 10^−7^ to 5.33 × 10^−7^) (He et al. 2013) and *Mycobacterium tuberculosis* (8.0 × 10^−8^ to 1.25 × 10^−7^) (Bryant et al. 2013), gram-positive bacteria which can undergo periods of dormancy and long-term persistence. The analysis provided a median estimate for the time of the most recent common ancestor (tMRCA) of our global sample of *S. equi* to 1909 (95% HPD: 1819 to 1946). Given that the historical record of strangles suggests that *S. equi* dates back to at least the 13th Century, our data indicate that a global population replacement occurred during the 19th or early 20th Centuries, corresponding to a time when horses were a major mode of transport and played important roles in a number of global conflicts (Fig. 3). To ensure this result was not an artifact of the clock or population models used in our analysis, we calculated the mean, median, and 95% HPD tMRCA for a range of model combinations and found that our chosen model exhibited the widest HPD, which encompassed the 95% HPDs of all other model combinations (Fig. 3).

To test our hypothesis that persistent isolates exhibited increased substitution rates, we performed a two-sided Mann-Whitney *U* test of median substitution rates for branches in our BEAST tree leading to persistent isolates against those leading to isolates from acute infection (Supplemental Fig. 6a). We found a significant difference (*P* = 0.0004) in rates between the two populations, with persistent isolates exhibiting a significantly increased rate. A linear regression of substitution rate against time for persistent, acute, and other (where the site was unknown) branches showed an increasing rate over time for persistent isolates (*R*^2^ = 0.20156, *P* < 0.0001), whereas the rates for acute (*R*^2^ = 0.01459, *P* = 0.3693) and other (*R*^2^ = 0.00323, *P* = 0.3815) branches showed no increase with time (Supplemental Fig. 6b). A similar disparity was identified in the types of substitutions occurring in acute and persistent infection, with more nonsynonymous mutations accruing over time in persistent infection when compared with isolates from acute infection and others (Supplemental Fig. 7a). In fact, whereas acute infection and other branches showed a relatively constant *d*_N_/*d*_S_ ratio of around 0.5–0.7 over time, persistent isolates showed an increase in *d*_N_/*d*_S_ with time (Supplemental Fig. 7b), supporting the hypothesis that these isolates are undergoing diversifying selection.

### Variation in the accessory genome was not associated with persistence or infection

Having observed distinct differences in the mutation rates of core genomes of the persistent and acute populations, we undertook a comparative genomic analysis to investigate if there were also differences in the dynamics of the accessory genome. Among the mobile elements described in the reference *Se*4047 isolate, the prophages φSeq2-4, and the integrative conjugative elements ICE*Se1* and ICE*Se2* were the most stable, being almost ubiquitously conserved in the population (Supplemental Fig. 8). In contrast, the prophage φSeq1 appeared more dynamic, with evidence of alternative but related prophages in the accessory genomes of isolates belonging to all four clusters. Although the pattern of presence of these prophage elements generally followed the phylogeny, there was evidence of lateral transfer or convergent gain of some prophages in multiple clusters. The deletion of an ancestral CRISPR locus, prior to the emergence of *S. equi* from *S. zooepidemicus,* potentially contributes to the poly-lysogeny of this species (Holden et al. 2009; Waller and Robinson 2013).

Although φSeq2-4, ICE*Se1*, and ICE*Se2* were generally conserved (Supplemental Fig. 8), we found evidence of both deletions and duplications affecting regions of these elements. Several isolates showed duplications of the phospholipase A_2_ gene, *slaA,* on φSeq2, suggesting that these strains had acquired additional distinct prophages that also contain this putative virulence cargo (Holden et al. 2009). Convergent loss of the *seeL* and *seeM* superantigen-encoding genes in six isolates from three distinct lineages appears to have occurred twice by loss or replacement by diverse alternative prophages of φSeq3, as evidenced by only <25% of φSeq3 having matches in the genome sequences of the isolates (Supplemental Fig. 8), and once by horizontal replacement of φSeq3 with an alternative, similar prophage, as evidenced by 60%–70% of φSeq3 having matches in the genome sequence of the isolates (Supplemental Fig. 8) and an elevated SNP density (mean of 1 SNP every 168 matched bases versus 1 SNP every 6130 matched bases for other isolates with at least one SNP).

### Insertion sequence elements mediate genome decay and gene amplification during persistence

Although our comparative analysis revealed that, as would be expected, prophages were mediating the loss and gain of genes, it also revealed that genes were being lost and duplicated in the core genome. The mechanism that appears to be driving the observed deletion and copy number variation is homologous recombination between insertion sequence (IS) elements. The *S. equi* genome has large expansions of a limited number of IS families, which is hypothesized to result from the evolutionary bottleneck associated with its host-adaptation and pathogenic specialization (Holden et al. 2009). We identified an additional 37 novel IS element insertions belonging to existing families across the population, eight of which inserted at the same genomic location in multiple lineages (Supplemental Figs. 9, 10). IS elements flanked four of the 15 deleted loci and 10 of the 16 amplified regions in the core genome, showing that they play an important role in variation of gene content.

**Figure 4.**
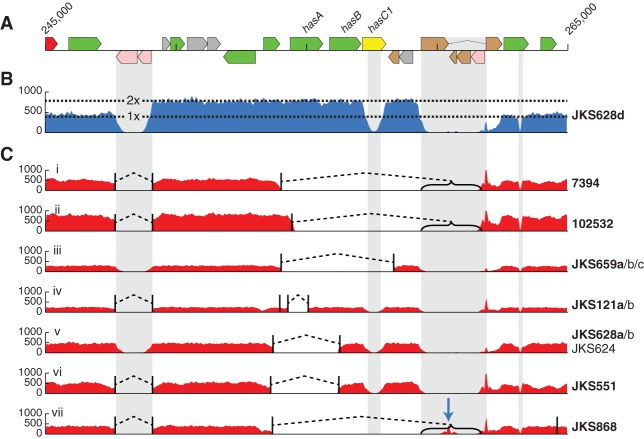
Convergent deletion and amplification of the *has* locus in isolates recovered from independent persistently infected horses. (*A*) A representation of the genome annotation around the *has* locus. (*B*) Eight isolates, all from different horses, independently exhibited duplications of the region between IS repeats (indicated by gray columns). This plot represents mapped sequence read coverage of JKS628d, showing an increase in coverage spanning the *has* operon, representing duplication of the region. Read mapping reduction in the gray repeat regions is due to an inability to map reads uniquely to those regions. (*C*) Twelve isolates from eight horses exhibited seven independent deletions of different regions of the *has* locus. Plots *i*–*vii* show read mapping of the *has* locus in example isolates for each of the deletions. Names to the *right* of the plots indicate isolates exhibiting the same deletion. Isolate names in bold are the examples used for the mapping plot. Read mapping drops to zero in the gray repeat regions due to an inability to map reads uniquely to this region. Deletion breakpoints, identified by finding breakpoints in reads, are indicated by vertical black lines joined by dashed bridges. Brackets indicate where one breakpoint location is uncertain due to falling in a repetitive region. The blue arrow in *vii* indicates the location of a novel IS insertion.

Consistent with our hypothesis that the *S. equi* genome diversifies within the guttural pouch environment, large deletion and duplication events were far more common in persistent isolates than those from acute infection (Supplemental Fig. 9). Within the core genome, the *has* operon, encoding hyaluronic acid capsule biosynthesis components, was the locus most frequently affected by deletions and duplications, with seven independent deletion variants and eight independent amplifications of the locus, which is bordered by IS*3* elements (Fig. 4). The same region also exhibited high SNP densities and four independent nonsense mutations. Importantly, the SNPs, deletions and amplifications at this locus were significantly associated with isolates recovered from persistently infected horses rather than those with acute disease (*P* < 0.0001). To determine the effects of these changes on the production of the hyaluronic acid capsule, we quantified the transcription across the *has* locus and the amount of hyaluronic acid present on the surface of five representative strains. The genome of strain *Se*4047 has been sequenced to completion and was included as a reference (Holden et al. 2009). Strain JKS551, recovered from a persistently infected horse, contains a deletion of *hasA* and the 3′ region of *hasB* (Fig. 4) relative to strain JKS540a, which was recovered from a horse suffering from acute disease during the same outbreak of strangles as JKS551. Reverse transcription qPCR confirmed a lack of *hasA* transcription in strain JKS551 (*P* = 0.0017), and this strain had significantly less hyaluronic acid associated with its cell surface (*P* = 0.04) (Supplemental Fig. 11). Strain 851, which contains an amplification of the *has* locus, had significantly higher levels of transcription of the *has* genes (*P* = 0.005, *P* = 0.0001, and *P* = 0.001 for *hasA*, *hasB*, and *hasC*, respectively) and a corresponding increase in the amount of hyaluronic acid on its surface (*P* = 0.0005) relative to strain 1165, which was recovered from the same persistently infected animal 2 wk later and contains a wild-type *has* locus (Supplemental Fig. 11). A specific PCR that spanned the IS*3* element that borders the *has* locus was used to confirm that the amplification of the *has* locus in strain 851 occurs in tandem.

**Figure 5.**
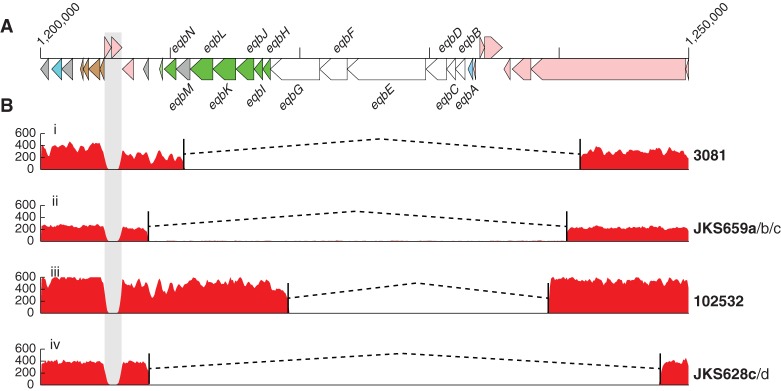
Convergent deletion of the equibactin locus in seven isolates recovered from four independent persistently infected horses. (*A*) A representation of the genome annotation around the equibactin cluster. (*B*) Read mapping of the equibactin locus in example isolates exhibiting the four deletion variants. Names to the *right* of the plots indicate isolates exhibiting the same deletion. Isolate names in bold are the examples used for the mapping plot. Read mapping drops to zero in the gray repeat region due to an inability to map reads uniquely to this region.

Comparison of the multiple isolates recovered from persistently infected horses showed that even these extremely closely related isolates varied by deletions and/or amplifications (Supplemental Fig. 12), demonstrating that microevolution of *S. equi* in the guttural pouch yields a mixture of variant strains. One example is the horse *JKS628*, within which two of the four persistent isolates, JKS628c and JKS628d, recovered from the right and left guttural pouches, respectively, contained a 39.5-kb deletion (SEQ_1232 to SEQ_1253) in ICE*Se2* that included the entire equibactin locus. This locus encodes a putative siderophore, which is known to increase the ability of *S. equi* to acquire iron (Heather et al. 2008), and may enhance survival within, and abscessation of, lymph nodes (Holden et al. 2009). This siderophore has been proposed to represent the key speciation event in the evolution of *S. equi* (Holden et al. 2009). Three other deletion events (Fig. 5) that are predicted to disrupt equibactin production were identified in isolates recovered from three more, unrelated persistently infected horses. In contrast, the equibactin region was not deleted in any of the acute isolates (*P* = 0.0528), suggesting that loss of equibactin may preclude transmission to acute infection.

**Figure 6.**
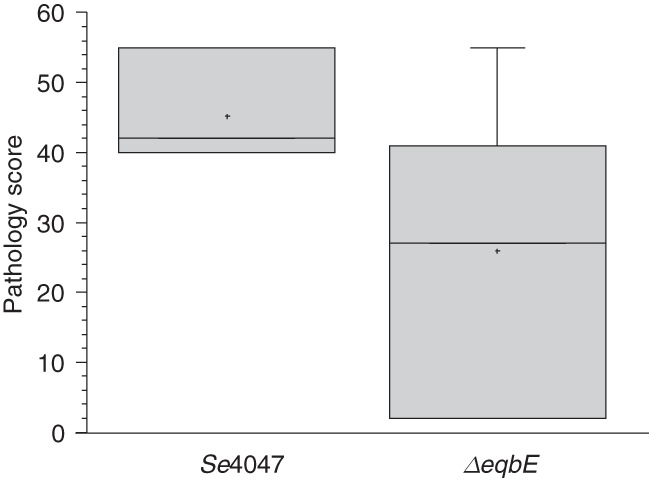
Pathology score of two groups of seven Welsh mountain ponies challenged with wild-type *Se*4047 or the Δ*eqbE* deletion mutant. The box illustrates the 50% distribution, the line indicates the median value, the cross indicates the mean, and error bars indicate the maximum and minimum distribution.

To determine if the loss of the equibactin locus represented a dead-end mutation that would prohibit these isolates from being able to cause acute disease and therefore continue to be transmitted via the strangles disease cycle, we challenged two groups of seven Welsh mountain ponies with 1 × 10^8^ cfu of *Se*4047 or an *eqbE* deletion mutant, which is unable to produce equibactin (Heather et al. 2008). Ponies were monitored carefully for early signs of disease, such as pyrexia and a preference for hay over pelleted food, and euthanized before the onset of later clinical signs of strangles (Guss et al. 2009). Deletion of *eqbE* significantly reduced the amount of pyrexia (*P* = 0.021) and pathology at post mortem examination (*P* = 0.041) (Fig. 6). However, loss of *eqbE* did not completely prevent abscess formation with two Δ*eqbE*-infected ponies developing bilateral retropharyngeal lymph node abscesses compared with all seven wild-type infected animals (*P* = 0.021). One Δ*eqbE*-challenged pony developed a submandibular lymph node abscess, two ponies had microabscesses without clinical signs of disease, and two had no signs of infection. The attenuation of the Δ*eqbE* deletion mutant in Welsh mountain ponies demonstrates that the acquisition of ICE*Se2* was indeed a key step in the evolution of *S. equi* and that strains containing deletions in the equibactin locus, which are shed from persistently infected horses, have a lower virulence potential.

## Discussion

For the first time, analysis of a global collection of *S. equi* isolates has allowed important questions regarding the complex epidemiology of strangles outbreaks to be resolved and provided compelling evidence of coinfection of individual horses. Variation between isolates in the collection was strikingly low given that the historical record of strangles suggests *S. equi* dates back over 700 yr. Our data indicate that a global population replacement occurred during the 19th or early 20th Centuries, corresponding to a period of history during which horses were, for the most part, the major mode of transportation and performed critical roles in a series of global conflicts (Clabby 1976). Strangles was, and remains, a particular problem in army horses (Todd 1910; Bazeley 1940). Indeed, the Leicestershire outbreak represented in this study that included horse *JKS121* occurred at the Defense Animal Centre in Leicestershire. Large global conflicts, such as World War I, led to the mobilization and mixing of large populations of horses from around the world in conditions ideally suited to the transmission and mixing of *S. equi* clones. In conjunction with this, dramatic levels of host mortality would have increased pathogen population bottlenecks and the potential for loss of population diversity. Later replacement of lost horses, through initiatives such as the establishment of the National Stud in the United Kingdom in 1916 for the breeding of thoroughbreds, produced naïve young horses on an unprecedented scale, providing new hosts for successful *S. equi* clones to colonize. Thus, we speculate that the unique circumstances created by 19th and early 20th Century global conflicts would have provided ideal conditions for the emergence of the contemporary population of *S. equi*.

The crucial factor allowing *S. equi* to propagate the disease cycle of strangles is its ability to persist for long periods in a subclinical but infectious state. The importance of the establishment of persistent infections to the capability of *S. equi* to transmit to naïve animals was first recognized by Captain Todd of the British Army Veterinary Corps as early as 1910 (Todd 1910) and is undoubtedly a major factor in its continued global success. However, it was not until 1997 that the anatomical site of such persistent infections was shown to be the guttural pouch (Newton et al. 1997). Our data suggest that the environment within the guttural pouch, and the chondroid in which *S. equi* persists, drives both the diversification and decay of its genome and potentially explains the path to host restriction. Although adaptation to the guttural pouch environment, such as loss of the equibactin siderophore, may reduce virulence and transmissibility, leading to an evolutionary dead end, it is clear that strangles outbreaks can be founded by long-term persistent strains. Indeed, persistence may also bring beneficial effects beyond simply providing time for contact with new naïve hosts. For example, mutation of genes encoding the antigenic, sortase-processed surface proteins SeM, SzPSe, and SEQ_0402 during persistence could assist the evasion of acquired immunity facilitating persistence in the guttural pouch and potentially the recrudescence of strangles. Increased shedding and/or more efficient transmission of new variant strains in this manner provides one explanation for the raised SNP densities observed in the genes encoding these proteins and highlights further genes that exhibit similar raised SNP densities, such as *fneE*, which will direct the design of improved vaccines against strangles. An interesting observation from the whole-genome phylogeny is that the increasingly prevalent ST151 clone of *S. equi* is found in a distinct clade to both the Equilis StrepE and Pinnacle IN vaccine isolates. The first identified isolates of ST151 were recovered from three horses with strangles <3 mo after they were vaccinated with Equilis StrepE (Kelly et al. 2006), raising the prospect that this lineage may escape current vaccines and should be closely monitored.

The loss and inactivation of genes required for hyaluronic acid capsule biosynthesis exclusively in isolates of *S. equi* recovered from persistently infected horses is paralleled by the loss of these genes in human disease isolates of *S. pyogenes* serotype M4 and M22 (Flores et al. 2012) and the presence of SNPs in the *has* locus of isolates recovered from cases of pharyngitis rather than invasive disease caused by *S. pyogenes* serotype M3 (Shea et al. 2011). Our data highlight similarities between these two streptococcal species and provide further evidence that *S. equi* infection can serve as a model for human disease. The tandem amplification of the *has* locus led to increased transcription of *hasA*, *hasB*, and *hasC* and significantly more hyaluronic acid on the surface of strain 851 relative to strain 1165, which was recovered from the same horse 12 d later. Theoretically the recombination of tandemly arranged amplified loci permits the restoration of a wild-type phenotype. This scenario could explain why later isolates from this animal (2424 and 3446) also contained a wild-type *has* locus. However, we cannot rule out the possibility that the population of *S. equi* from this horse contained more than one *has* variant on each occasion. We propose that the amplification of the *has* locus may assist *S. equi* to establish a persistent infection within the guttural pouch in the immediate period post-recovery when the equine immune response may be at its most effective. However, we speculate that the restoration of a wild-type phenotype or the loss of capsule production may be favored over time during persistent infection, possibly as chondroids form, nutrient levels decline, or the immune response becomes gradually less effective. We speculate that amplifications of loci, including *has*, may be present in isolates of *S. pyogenes* recovered from cases of pharyngitis, and this strategy provides a novel bet-hedging solution to survival in fluctuating environments.

The lifecycles of many pathogens involve both acute and persistent phases of infection. Balancing the conflicting genetic requirements of transmission and clinical disease with survival in the face of a convalescent immune response represents a significant biological challenge that may ultimately lead toward host restriction. By sequencing multiple isolates from the same infected horses our analyses have, for the first time, shed light on the complex population dynamics of *S. equi* during persistence within the host. The observation of an increased substitution rate within persistent infection mirrors the situation in human immunodeficiency virus (HIV) infection where mutations accrue faster within hosts than at the epidemic level (Herbeck et al. 2006; Lemey et al. 2006). Mathematical modeling of this phenomenon supports the hypothesis that ancestral HIV sequences are preferentially transmitted, purifying out mutations generated within the host and reducing the apparent substitution rate at transmission (Lythgoe and Fraser 2012). Our data suggest a similar process has shaped the evolution of *S. equi*, and may be important in all pathogens with a lifecycle which includes long periods of infection between transmission events. For example, clear parallels can be drawn with the discrepancy between the slow observed substitution rate in *M. tuberculosis* transmission chains (Bryant et al. 2013; Walker et al. 2013) and its ability to quickly develop antibiotic resistance mutations within a host (Sun et al. 2012; Casali et al. 2014). For *S. equi*, the ability to adapt and persist in healthy animals for long periods while remaining infectious is a crucial component of the disease cycle of this pathogen. The fluidity of its genome plays a key role in ensuring *S. equi* remains a persistent threat to equine health with the mobility of modern horse populations facilitating its transmission around the world.

## Methods

### Study collection

*Se*4047 was isolated from a horse with strangles in the New Forest, Hampshire, United Kingdom, in 1990. The origins and details of the 224 isolates of *S. equi* that were sequenced in this study are listed in Supplemental Table 1. Isolates from acute cases of strangles were recovered from clinical samples submitted to the Animal Health Trust's Diagnostic Laboratory Service citing clinical signs that were indicative of acute disease or were recovered during veterinary examination of acute clinical cases in the United Kingdom, Sweden, Belgium, or Saudi Arabia. Persistent isolates were recovered from the guttural pouches of horses exhibiting no clinical signs of infection, from chondroids recovered from a guttural pouch, or from swabs of which the attending veterinarian stated that the animal was suspected to be persistently infected. β-Hemolytic colonies of *S. equi* strains were recovered from glycerol stocks following overnight growth on COBA strep select plates (bioMérieux). Their identity was confirmed by a lack of fermentation of trehalose, ribose, and sorbitol in Purple broth (Becton Dickinson).

### DNA preparation

A single colony of each *S. equi* strain was grown overnight in 3 mL Todd Hewitt (TH) broth containing 30 µg/mL hyaluronidase (Sigma) at 37°C in a 5% CO_2_-enriched atmosphere, centrifuged, the pellet resuspended in 200 µL Gram +ve lysis solution (GenElute, Sigma) containing 250 units/mL mutanolysin and 2 × 10^6^ units/mL lysozyme and incubated for 1 h at 37°C to allow efficient cell lysis. DNA was then purified using GenElute spin columns according to the manufacturer's instructions (all Sigma).

### DNA sequencing and variation detection

Library construction for Illumina sequencing was carried out as described previously (Quail et al. 2012). One isolate, NCTC9682, was sequenced on an Illumina GAII for 54 cycles from each end, producing paired-end reads with an expected insert size of 250 bp. The remaining isolates were mixed in pools of between 20 and 24 to produce multiplexed libraries that were sequenced on the Illumina HiSeq platform for 75 cycles from each end plus an 8-base index-sequence read. Again, the expected insert size between the paired-end reads was 250 bp. Short reads were mapped against the reference *Se*4047 genome (accession numbers: FM204883 and NC_012471) using SMALT v0.7.4 (https://www.sanger.ac.uk/resources/software/smalt). Locations of deletions and short insertions were predicted using Pindel (Ye et al. 2009) and then validated by comparing the mapping of reads spanning indels to the reference genome and to a version of the same reference with the predicted indel included. If the inclusion of the indel improved mapping, that indel was retained and reads realigned around it as per the remapping. SNPs were identified using a combination of SAMtools (Li et al. 2009) mpileup and BCFtools as previously described (Harris et al. 2010). Large deletions and duplications were identified for each isolate based on read coverage along the reference genome using a continuous hidden Markov model with three states: 0× coverage, 1× coverage, and ≥2× coverage. Initial and transition frequencies were fitted to the data using a Baum-Welch optimization, and the most likely sequence of hidden states was calculated using the Viterbi algorithm (Viterbi 1967). IS element insertion locations were identified by mapping read data to a set of IS sequences known to occur in *S. equi* and *S. zooepidemicus*. Where one read of a pair mapped to an IS element, the nonmapping paired read was mapped to the reference *Se*4047 genome to identify the insertion site. To remove false positive insertion sites that may be caused by chimeric read pairs, a minimum of five reads were required to map to the same insertion location for it to be accepted.

### Phylogenomic analysis

Maximum likelihood phylogenetic reconstruction of variable sites was performed using RAxML v7.0.3 (Stamatakis 2006) under a general time reversible (GTR) evolutionary model using a gamma correction for among-site rate variation. One hundred random bootstrap replicates were run to provide a measure of support for relationships in the maximum likelihood tree. A linear regression of root-to-tip distance versus isolation date was used to assess the fit of a strict molecular clock to the data and gave a weak correlation for isolates with known isolation dates, excluding vaccine isolates (coefficient of determination, *R*^2^ = 0.25). To test the null hypothesis that such an *R*^2^ arose by chance alone, we repeated the root-to-tip analysis 1000 times with the tip dates of the isolates randomly permutated each time (Firth et al. 2010). In all cases, the data with random permutations gave lower *R*^2^ values than the real data, so that we could reject our null hypothesis at the 0.001 level and accept the alternative hypothesis that the real data contains a significant temporal signal. Bayesian reconstruction in BEAST v1.7 (Drummond and Rambaut 2007) was used to estimate substitution rates and times for divergences of internal nodes on the tree under a GTR model with a gamma correction for among-site rate variation. All combinations of strict, relaxed lognormal, relaxed exponential, and random clock models and constant, exponential, expansion, logistic, and skyline population models were evaluated. For each, three independent chains were run for 100 million generations, sampling every 10 generations. On completion, each model was checked for convergence, both by checking that ESS values were >200 for key parameters and by checking that independent runs had converged on similar results. Models that failed to converge, including logistic population models, were discarded. Models were compared for their fit to the data using Bayes factors based on the harmonic mean estimator as calculated by the program Tracer v1.4 from the BEAST package. The best-fit model combination was found to be a relaxed exponential clock model and a skyline population model, and so this combination was used for all further analysis. A burn-in of 10 million states was removed from each of the three independent runs of this model before combining the results from those runs with the logcombiner program from the BEAST package. A maximum clade credibility (MCC) tree was created from the resulting combined trees using the treeAnnotator program, also from the BEAST package.

### Accessory genome assembly

A pan genome for *S. equi* was created by identifying novel regions from de novo assemblies of each isolate. Assemblies were created with Velvet v1.2.09 (Zerbino and Birney 2008) using the VelvetOptimiser.pl v2.2.5 (http://bioinformatics.net.au/software.shtml) script to optimize the kmer length, expected coverage, and coverage cutoff parameters based on the N50 statistic. A core genome was defined by removing the mobile prophages, φSeq1-4, and ICE elements, ICE*Se1* and ICE*Se2*, from the *Se*4047 reference genome. This core genome was then mapped to each assembly using NUCmer (Delcher 2002). All regions >200 bp in each assembly that did not match to the core genome were extracted and retained as accessory regions. All accessory regions for each isolate and the accessory regions from the reference *Se*4047 genome were then mapped against one another using NUCmer. Where two regions were identical in length and matched along their entire length, one was retained. Where one region was completely contained within another, the longer region was retained. Any region of novel sequence >200 bp was also retained. Using this process for all pairwise comparisons led to production of a nonredundant set of accessory genome contigs. After filtering, these accessory contigs were appended to the core genome to form a pan genome. Finally, each assembly and the reference *Se*4047 genome were mapped to the pan genome using NUCmer to identify the regions of the pan genome present in each isolate.

### Mutation frequency

Cultures of test bacteria were grown overnight in TH broth at 37°C in a 5% CO_2_ enriched atmosphere. Cultures were diluted to an OD_600 nm_ of 0.5. The number of viable bacteria was enumerated by plating 100 µL of a 10^−6^ dilution onto each of five TH agar plates that were grown at 37°C in a 5% CO_2_ enriched atmosphere for 24 h. One hundred microliters of undiluted culture was plated onto each of five TH agar plates containing 0.03 µg mL^−1^ rifampicin and grown at 37°C in a 5% CO_2_ enriched atmosphere for 24 h. Colonies were enumerated, averaged, and the resistance frequency was calculated by dividing the total number of colonies by the number of rifampicin-resistant colonies. The experiment was repeated in triplicate, and an average resistance frequency was calculated across the three experiments. Statistical significance was calculated using an ANOVA test on the whole population.

### Extraction of RNA

*S. equi* strains were inoculated onto COBA plates and grown overnight at 37°C in an atmosphere containing 5% CO_2_. A single colony of each strain was inoculated into 5 mL Todd Hewitt broth containing 0.2% yeast extract (THY) (Oxoid) and grown overnight at 37°C in an atmosphere containing 5% CO_2_. Cultures were diluted 1:10 in fresh THY and grown to an OD_600 nm_ of 0.6. Two milliliters of each culture was then mixed with 4 mL of RNA protect (Qiagen) and pelleted by centrifugation for 10 min at 5000*g* and then 8000*g* for a further 10 min. Each pellet was resuspended in 200 µL of tris-EDTA buffer (TE) (Fluka) containing 3 mg of lysozyme (Sigma), 500 units of mutanolysin (Sigma), and 60 µg hyaluronidase (Sigma). Total RNA was extracted using RNeasy mini and DNase kits (Qiagen) according to the manufacturer's instructions with the following alterations. After incubation at room temperature for 45 min with repeated vortexing, 700 µL of RLT buffer from the RNeasy mini kit was added and the tube vortexed briefly. The lysis mixture was transferred to a tube containing 0.05 g of acid washed glass beads (Sigma) and vortexed constantly for 5 min. The lysis mixture was centrifuged for 10 sec at 16,100*g*, and the supernatant was removed for RNA extraction with an RNeasy mini kit. RNA purity and quantity were determined using a Nanodrop ND1000 spectrophotometer (NanoDrop Technologies). Three biological replicates were performed.

### Reverse transcription

One hundred nanograms of RNA per sample were used to synthesize cDNA by reverse transcription (RT) in 20 µL reactions with random hexamers using a Verso cDNA kit (Thermo Scientific) according to the manufacturer's instructions.

### qPCR to quantify *has* transcription

Transcription of SEQ_0269 (*hasA*), SEQ_0270 (*hasB*), SEQ_0271 (*hasC*), and the housekeeping gene *gyrA* (SEQ_1170) were quantified by quantitative polymerase chain reaction (qPCR). Reactions contained 1× Kapa SYBR Fast qPCR mix (Anachem), 0.3 µM forward and reverse primers (Supplemental Table 3), and 6 µL of a 1:10 dilution of cDNA made up to 20 µL with ddH_2_O and thermocycled for 3 min at 95°C followed by 40 cycles for 30 sec at 95°C, 10 sec at 60°C, then for 15 sec at 95°C. A ramp step from 60°C to 95°C with SYBR reads every 0.3°C was performed to calculate the dissociation curves of products. No template and no RT controls were included to confirm the absence of contaminating DNA and RNA in samples. Copy numbers were calculated from standard curves and normalized to *gyrA*. The mean values for the three biological replicates were calculated.

### PCR to detect tandem amplification of the *has* locus

PCR reactions contained 1× Sigma Taq buffer, 2 mM deoxyribonucleotide triphosphate (dNTP) mix, 2 mM MgCl_2_, 1 µM forward and reverse primers (Supplemental Table 3), 0.1 µL Sigma Taq DNA polymerase, and 4 µL DNA in a total volume of 20 µL. Reactions were thermocycled for 2 min at 95°C followed by 35 cycles for 30 sec at 95°C, 30 sec at 55°C and 5 min at 72°C, and finally for 5 min at 72°C. Products were resolved on a 0.7% agarose gel.

### Quantification of surface hyaluronic acid

*S. equi* strains were grown overnight in THY at 37°C in an atmosphere containing 5% CO_2_ and used to inoculate 10 mL of THY. Triplicate cultures were grown to an OD_600_ of 0.6 at 37°C in an atmosphere containing 5% CO_2_. Bacteria were washed twice with water, resuspended in 0.5 mL of water and hyaluronic acid released by vigorous shaking with 1 mL of chloroform for 15 sec. The mixture was allowed to stand at room temperature for 1 h before centrifugation at 16,000*g*. The aqueous phase was removed and the amount of capsule determined as described previously (Schrager et al. 1996; Darmstadt et al. 2000). DNA was extracted from 1.5 mL of the OD_600 nm_ 0.6 culture using the GenElute spin columns as described above. The DNA was quantified using a Nanodrop ND1000 spectrophotometer, and the number of bacteria was determined using the Life Technologies DNA copy number calculator (https://www.lifetechnologies.com/uk/en/home/brands/thermo-scientific/molecular-biology/molecular-biology-learning-center/molecular-biology-resource-library/thermo-scientific-web-tools/dna-copy-number-calculator.html). The amount of hyaluronic acid was expressed as femtograms per copy of bacterial DNA.

### Allelic replacement

The generation of the *eqbE* deletion mutant, Δ*eqbE*, has been described previously (Heather et al. 2008).

### Experimental infection of ponies

Ponies of 18 mo of age were transferred to a containment unit 3 d before challenge. Each pony was challenged with *S. equi* strain 4047 or the Δ*eqbE* deletion mutant via the spraying of a 2 mL culture containing 5 × 10^7^ cfu into each nostril. Bacteria were grown overnight in Todd Hewitt broth and 10% fetal calf serum (THBS) in a 5% carbon dioxide enriched atmosphere at 37°C, diluted 40-fold in fresh prewarmed THBS, further cultivated, and harvested at an OD_600 nm_ = 0.3. This infection dose has been shown to optimize the infection rate, while avoiding overwhelming the host immune response (Hamilton et al. 2006; Guss et al. 2009). Ponies were monitored for the onset of clinical signs of disease over a period of 3 wk post-challenge by daily physical examination, rectal temperature, lymph node swelling, and nasal discharge scoring. Blood samples were taken for quantification of fibrinogen and neutrophil levels by total white blood count performed on a Beckman-Coulter ACTdiff analyzer with a manual differential count to calculate the percentage of neutrophils. Postmortem examination was performed on all ponies following the onset of early clinical signs of infection such as pyrexia and a reluctance to eat dry pelleted food, preferring haylage or water, and prior to the onset of later signs according to strict welfare guidelines at the Animal Health Trust or on reaching the study endpoint at 3 wk post-challenge. The severity of disease pathology was quantified according to a scoring system described previously (Guss et al. 2009). This work was conducted under the auspices of a Home Office Project License and following ethical review and approval by the Animal Health Trust's Animal Welfare and Ethical Review Body (RPP 01_12).

## Data access

Short reads for all sequenced isolates have been submitted to the European Nucleotide Archive (ENA; http://www.ebi.ac.uk/ena/) under study accession number ERP000812. Individual accession numbers of sequences and assemblies for all isolates are listed in Supplemental Table 1.

## Supplementary Material

Supplemental Material
