# Supplementary material for: Genome specialization and decay of the strangles pathogen, Streptococcus equi, is driven by persistent infection
Source: Genome Res. 2015 Sep;25(9):1360–71. doi: 10.1101/gr.189803.115 (PMC4561494; doi:10.1101/gr.189803.115)
Supplement: Supplemental Material [file supp_25_9_1360__index.html]

Genome specialization and decay of the strangles pathogen, Streptococcus equi, is driven by persistent infection — Genome specialization and decay of the strangles pathogen, Streptococcus equi, is driven by persistent infection — Genome specialization and decay of the strangles pathogen, Streptococcus equi, is driven by persistent infection — Supplemental Material 

# Genome specialization and decay of the strangles pathogen, *Streptococcus equi*, is driven by persistent infection

## Supplemental Material

**Files in this Data Supplement:**

- Supp Figures.pdf
- Supp Tables.xlsx
