# Supplementary material for: Genome specialization and decay of the strangles pathogen, Streptococcus equi, is driven by persistent infection
Source: Genome Res. 2015 Sep;25(9):1360–71. doi: 10.1101/gr.189803.115 (PMC4561494; doi:10.1101/gr.189803.115)
Supplement: Supplemental Material [file supp_gr.189803.115_SuppFigures.pdf]

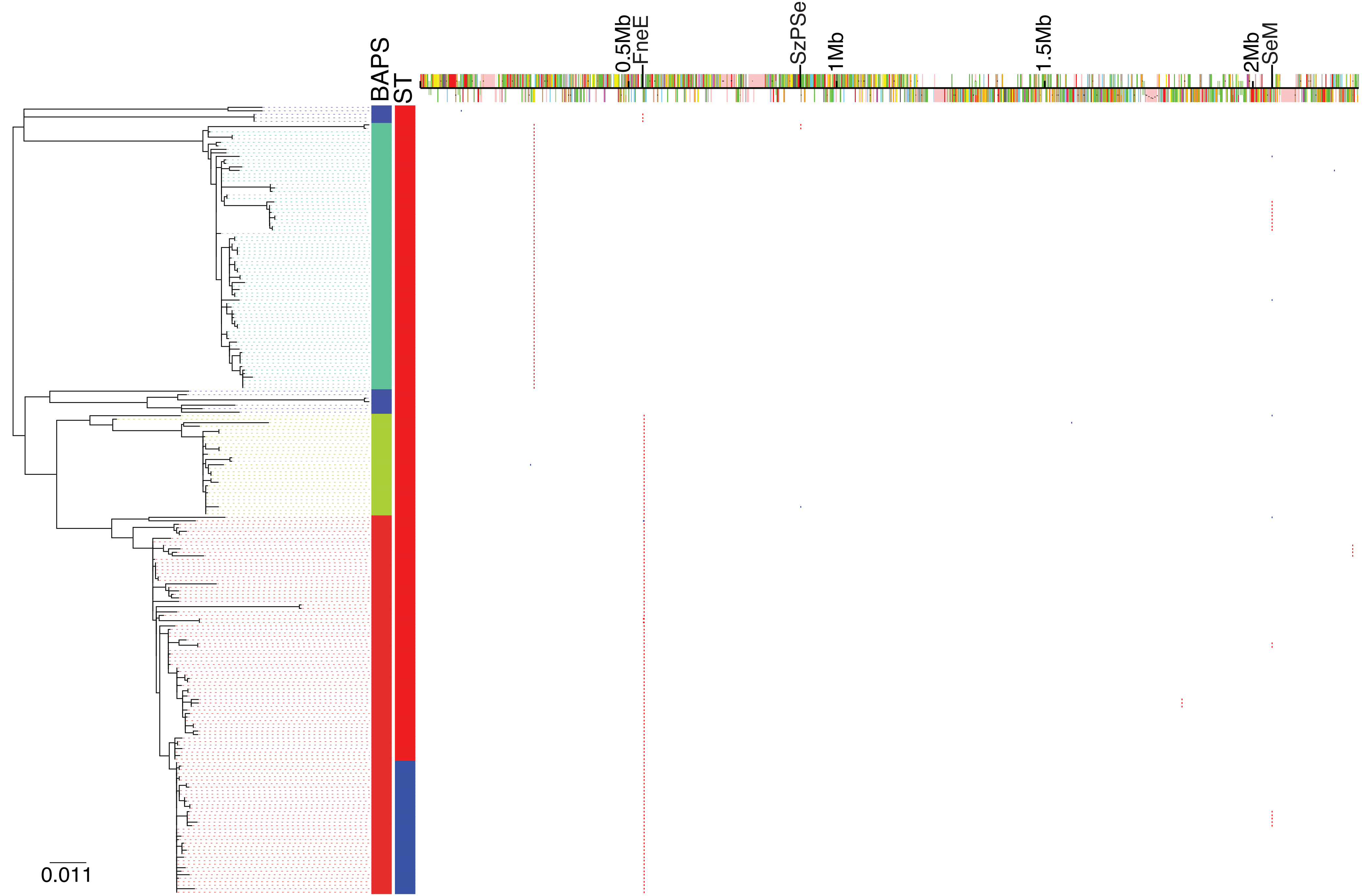

Supplementary Figure 1. Representation of predicted homologously-recombined regions. The left panel represents the ML phylogeny of *S. equi*, with BAPs cluster and MLST type shown in columns adjacent to the tree, as in Fig. 1. The right panel represents regions identified as exhibiting significantly raised SNP density indicative of import of variation *en masse* via homologous recombination or regions under high selective pressures. Above the panel is a representation of the genome annotation of *Se*4047 with the locations of the genes encoding SeM, FneE and SzPSe indicated.

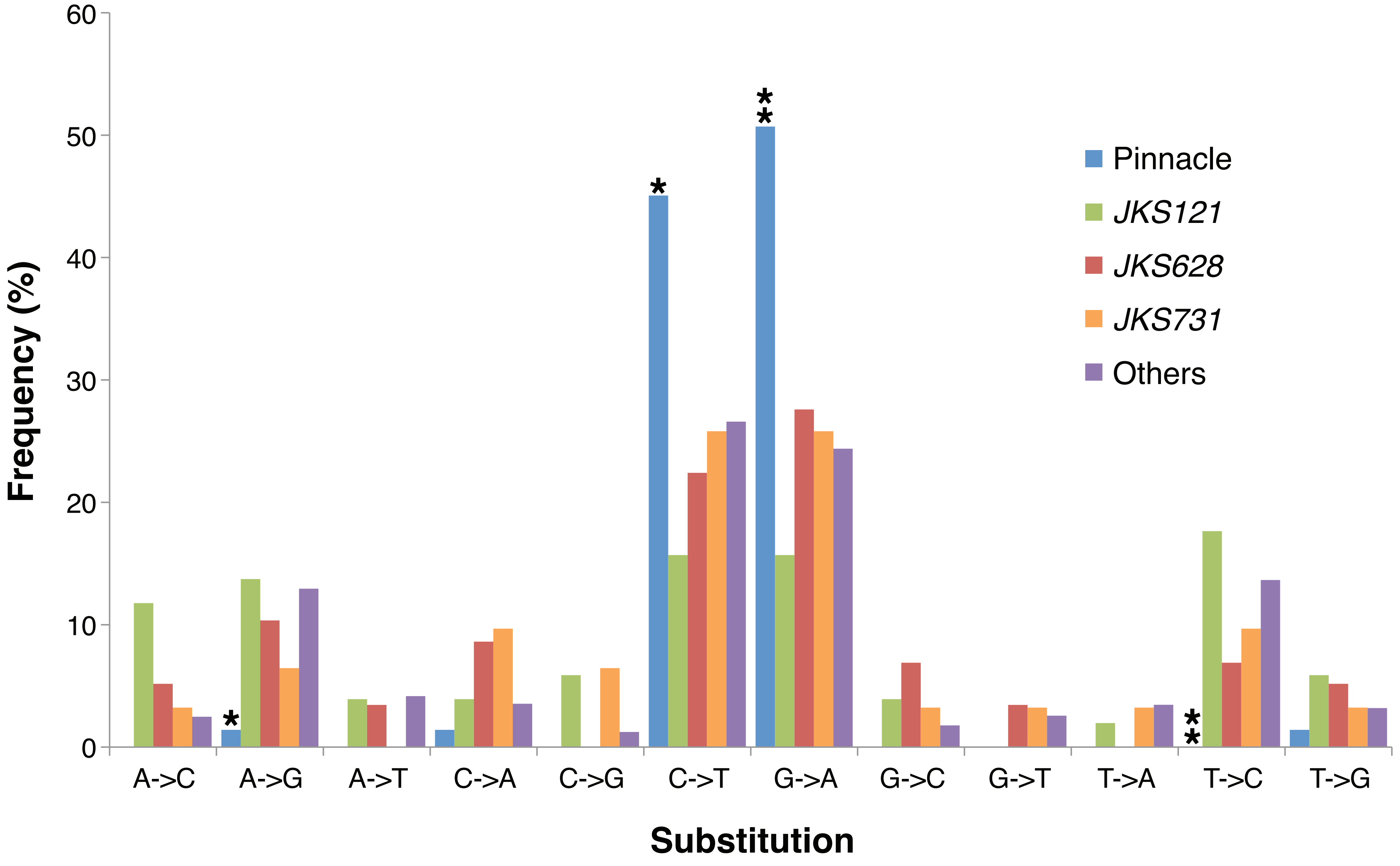

Supplementary Figure 2. Mutation spectra associated with branches on the tree leading to the outliers in the root-to-tip analysis (Fig. 1) and all other branches. * indicates significant difference to 'others' at the 0.1 level, while ** indicates significance at the 0.05 level. Colors correspond to colors in Fig. 1.

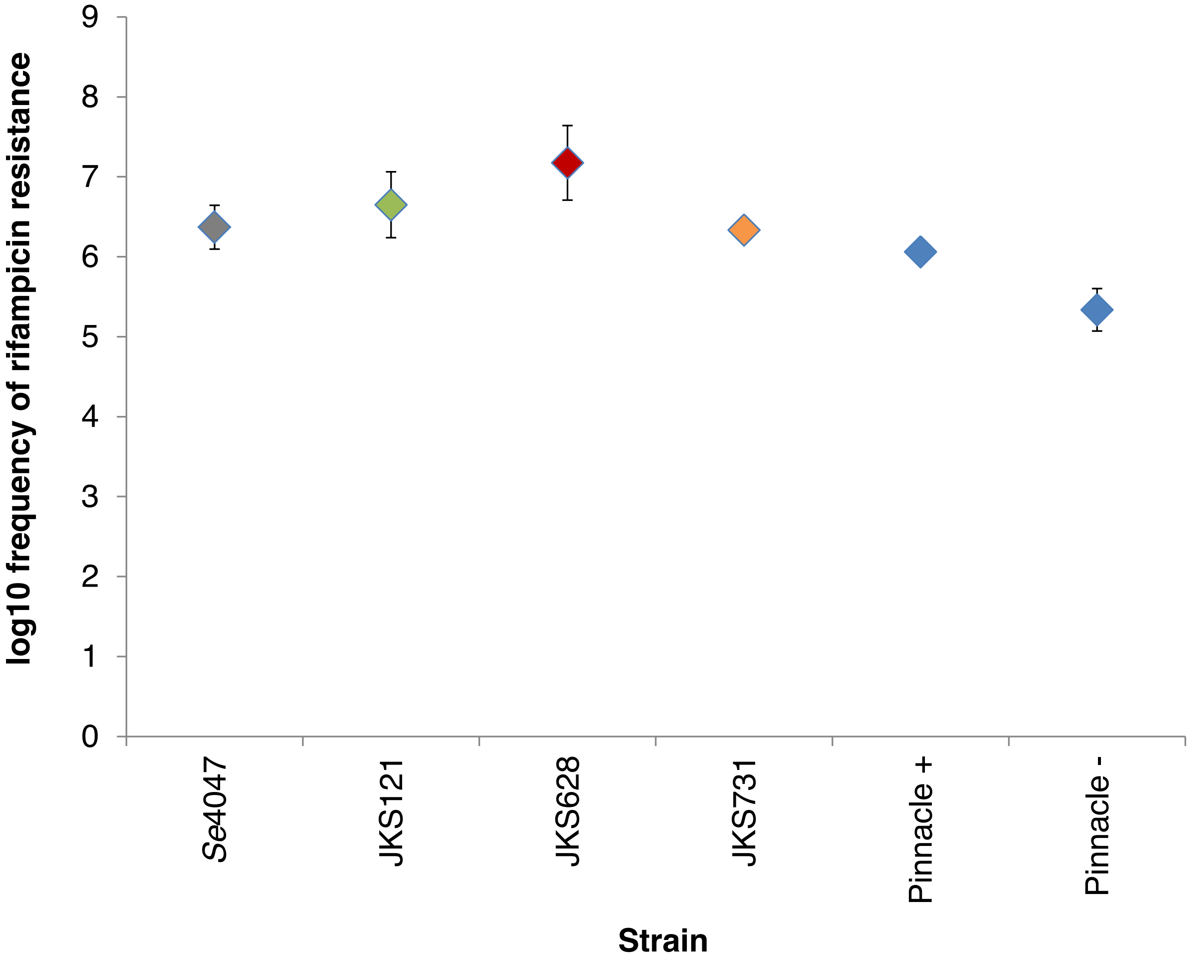

Supplementary Figure 3. Mean resistance frequency of long-branch isolates and the reference *Se*4047 *in vitro* to rifampicin. Values represent the means of three independent experiments conducted in triplicate. Error bars indicate 95% confidence intervals.

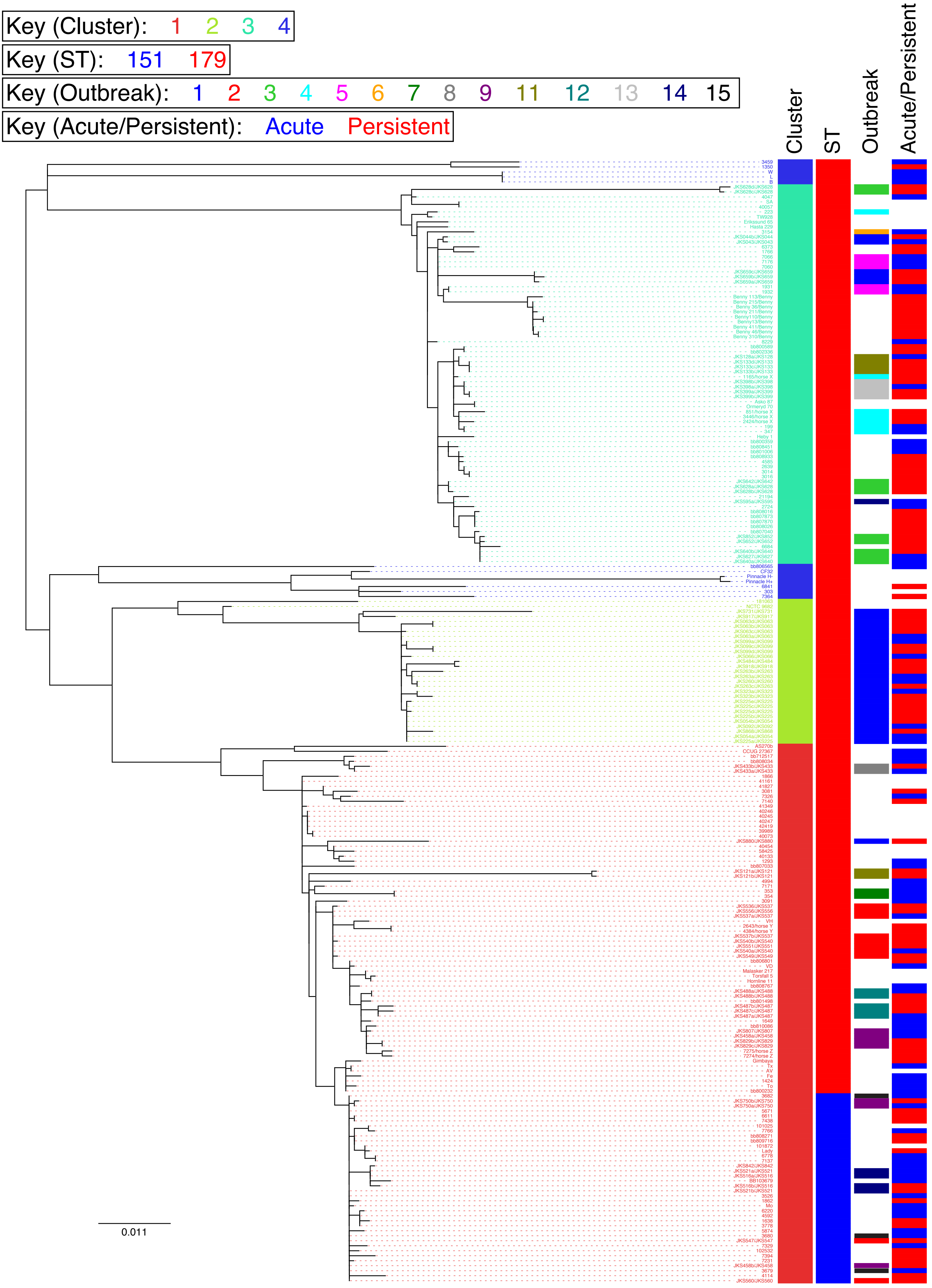

Supplementary Figure 4. Phylogenetic visualisation of isolate metadata. Labelled on the maximum likelihood phylogenetic reconstruction of *S. equi* are the BAPs cluster, sequence type (ST) and outbreak into which each isolate falls, and whether the isolate was from acute or persistent infection, as described in the Methods.

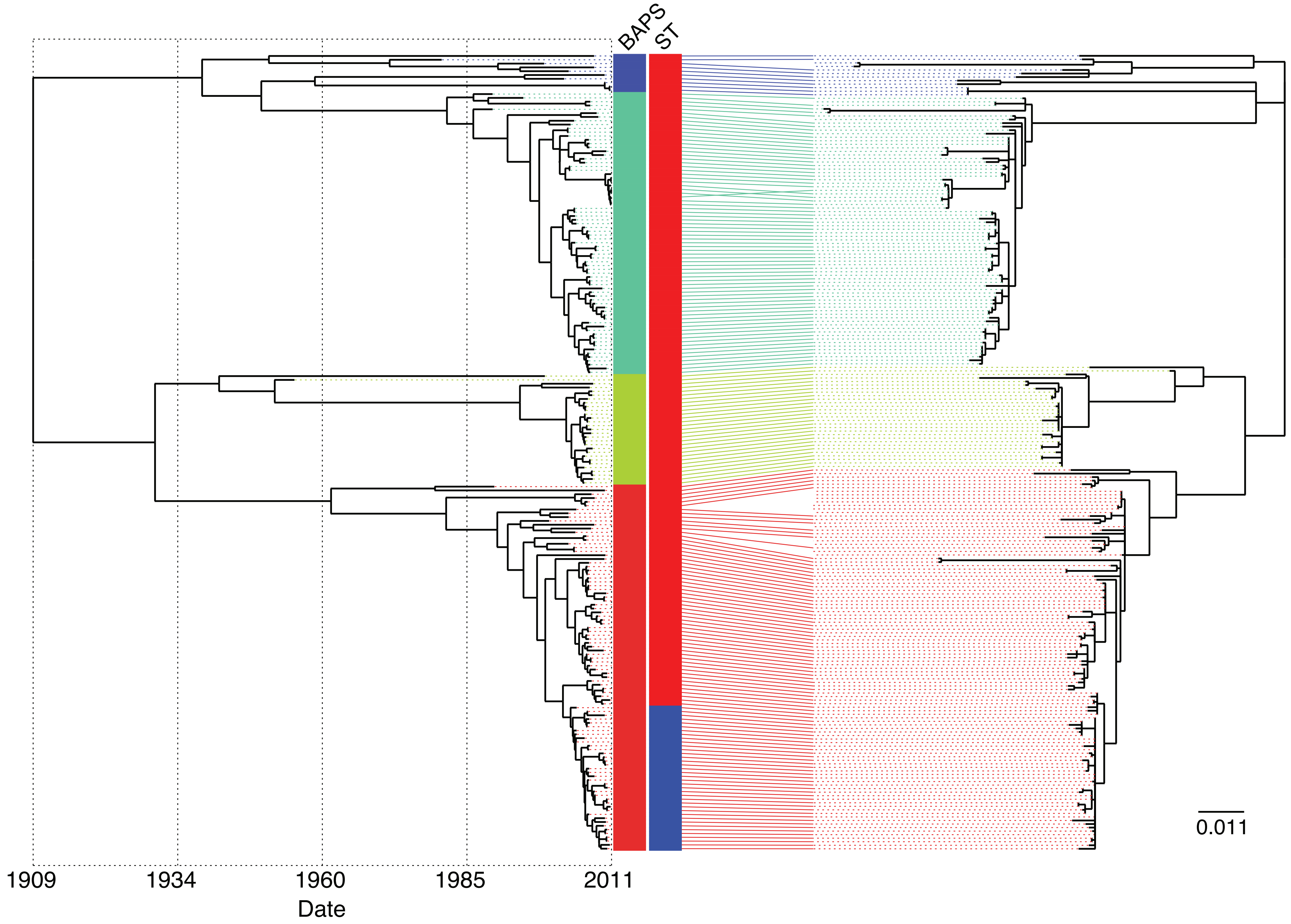

Supplementary Figure 5. Tanglegram showing concordance in BEAST (left) and ML (right) tree topologies, but not branch lengths. Branch lengths in the Bayesian phylogeny produced with BEAST represent time, while those in the ML tree represent genetic diversity. Dates are shown beneath the BEAST tree, and BAPs cluster and MLST type in columns adjacent to the tree.

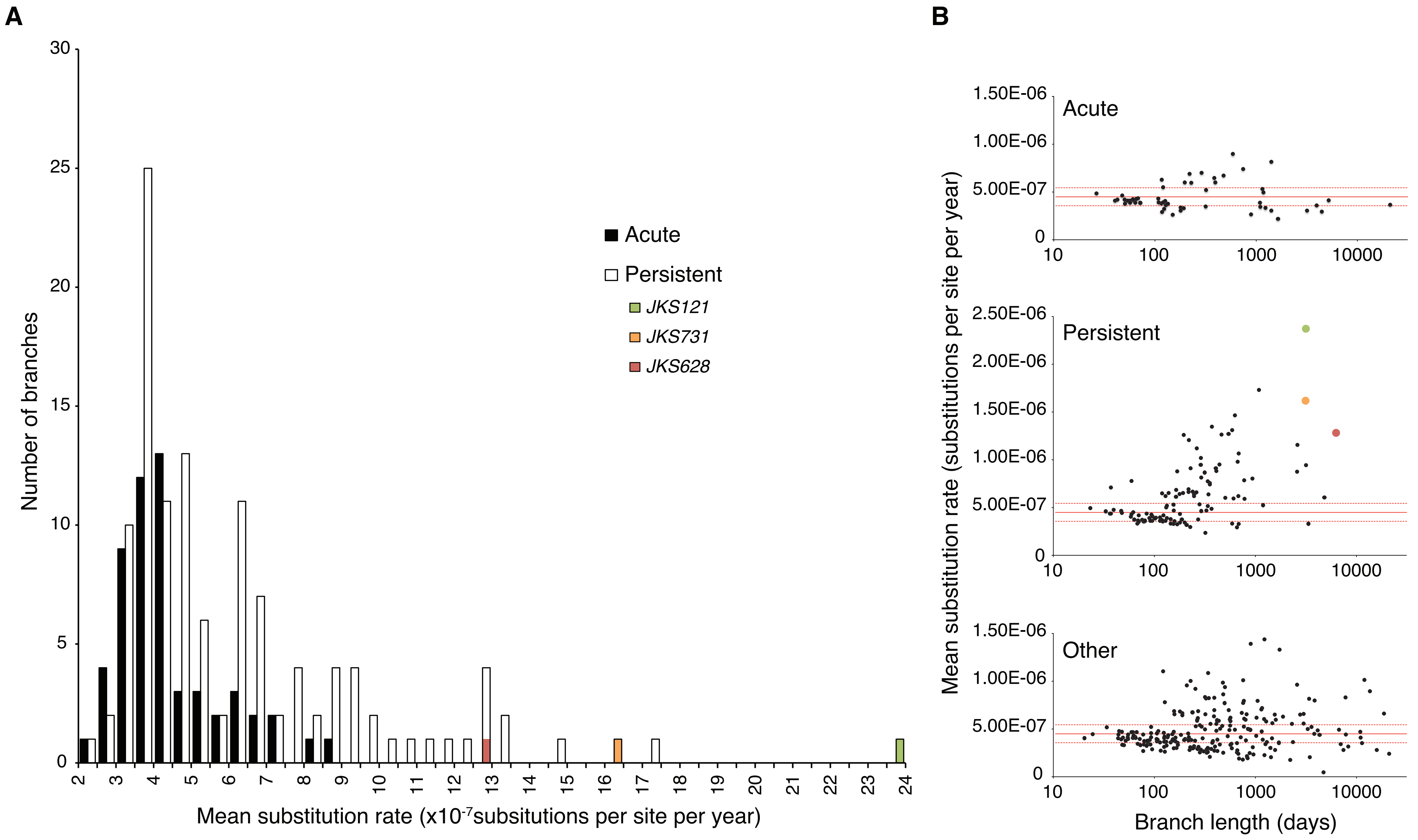

Supplementary Figure 6. Variation in substitution rates between acute and persistent isolates. A) Histogram showing the frequency of branches subtending acute and persistent isolates with different estimated mutation rates. The long branches subtending isolates from horses *JKS121*, *JKS628* and *JKS731* are colored using the same colors as used in Fig. 1. B) Scatter plots of branch length vs mean estimated substitution rate for branches subtending acute, persistent and other (unknown or mixed) isolates. The unbroken and dashed red lines in each plot indicate the mean and 95% HPD estimates for the entire data from BEAST. Enlarged, colored points correspond to the long branches subtending isolates of horses *JKS121*, *JKS628* and *JKS731*, as in part A.

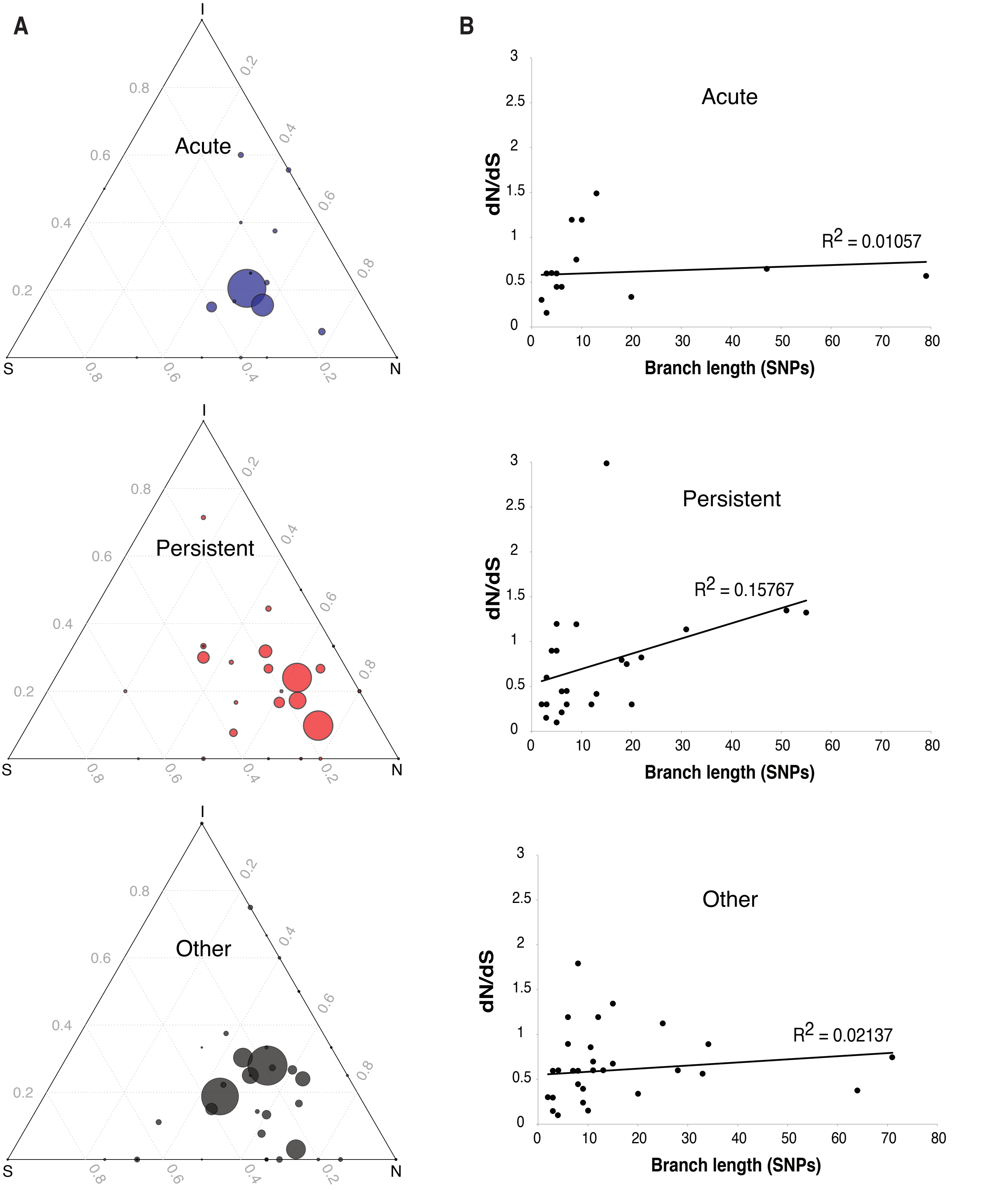

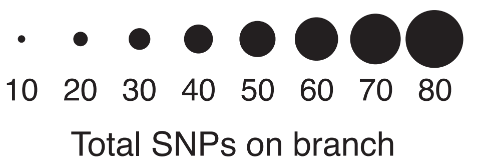

Supplementray Figure 7. A) Ternary diagrams showing the proportion of synonymous (S), nonsynonymous (N) and intergenic (I) SNPs on each branch of the *S. equi* phylogeny, as reconstructed by the deltran parsimony algorithm. Branches are split into three diagrams representing those subtending acute isolates, those subtending persistent isolates and others (unknown/mixed). The size of each point represents the total number of SNPs on the branch, as shown in the key. B) dN/dS vs branch length (in parsimony reconstructed SNPs) for branches subtending acute isolates, those subtending persistent isolates and others (unknown/mixed).

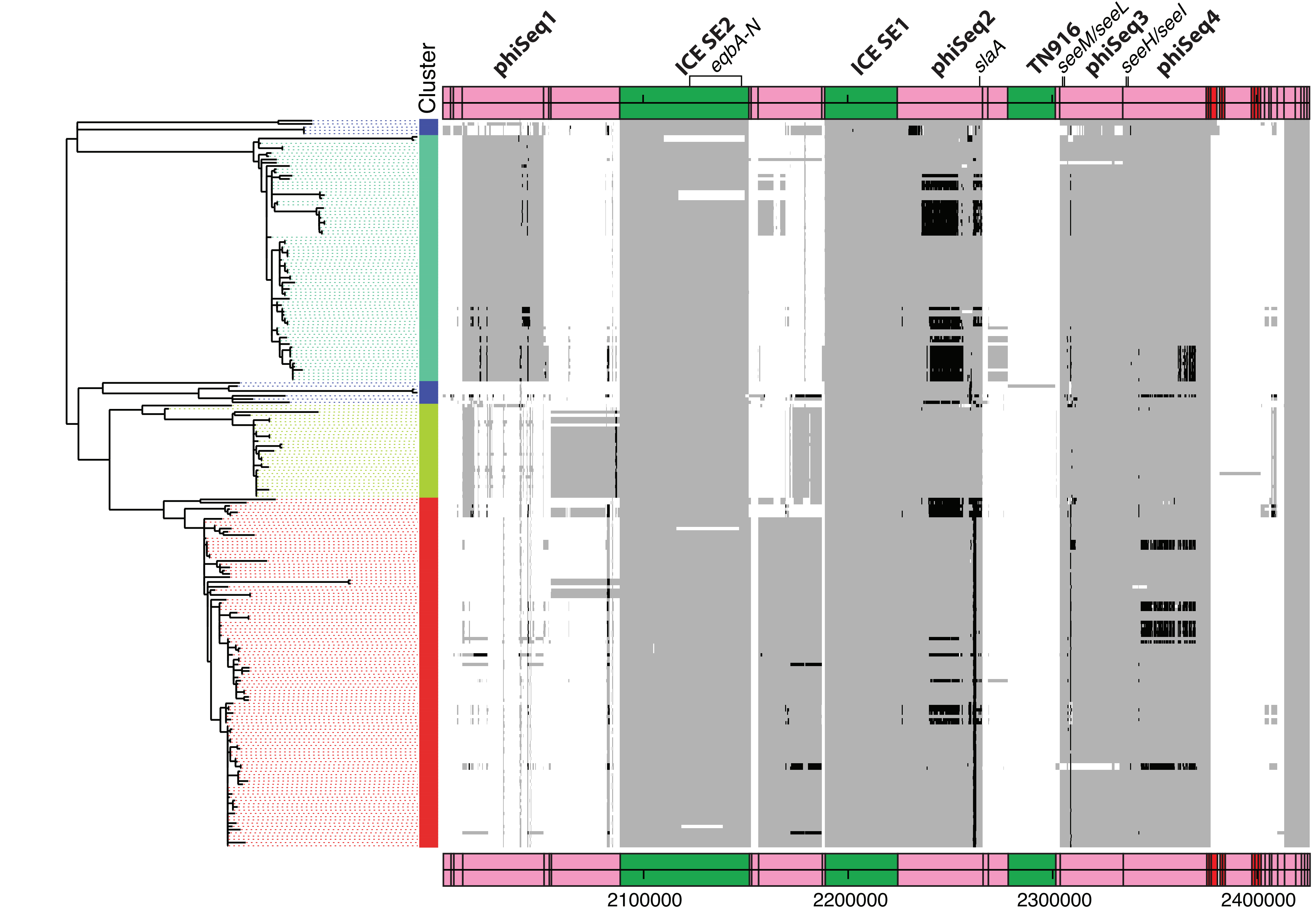

Supplementary Figure 8. Coverage of the accessory genome across the species. The left panel shows the ML tree, with BAPs cluster in a column to the right, as in Fig. 1. The right-hand panel shows coverage of the accessory genome in each isolate. To the top and bottom of the right panel are representations of the assembled accessory contigs from isolates in the study. The contig color gives an indication of the content of the contig. Pink = bacteriophage, green = integrative and conjugative element (ICE), red = IS element. Contigs present in the reference genome are labeled in bold above the panel, along with the location of some important virulence genes. For each isolate in the tree, regions are colored gray if they were present in single copy, and black if they were in multiple copy. i.e. duplications. Missing regions are in white.

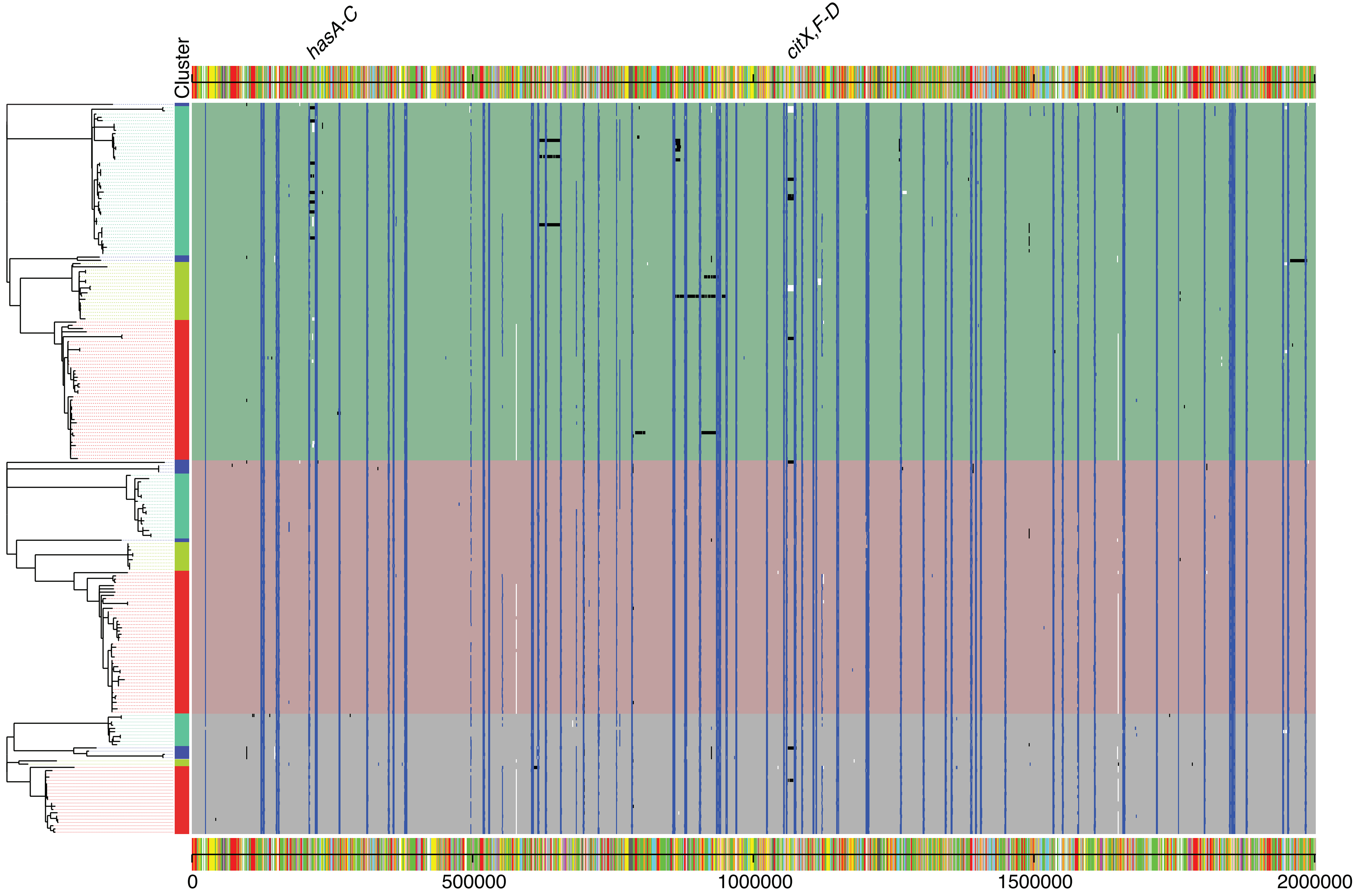

Supplementary Figure 9. Core genome insertions, duplications and IS elements in persistent, acute and other isolates. Three ML phylogenetic trees are shown in the left panel, created from only persistent isolates (top), only acute isolates (middle) and other isolates (bottom). For each, the column to the right of the tree indicates the BAPs cluster of the isolates in the species phylogeny in Fig. 1. In all cases, the topologies of the individual trees are consistent with the tree in Fig. 1. The right-hand panel shows coverage of the core genome in each isolate. To the top and bottom are a representation of the annotation of the core genome, with the *has* and *cit* loci labeled. Regions of single copy coverage are colored green in persistent isolates, red in acute isolates and gray in others. Regions of duplication are colored black, and deletions are white. IS element insertion locations are shown in blue.

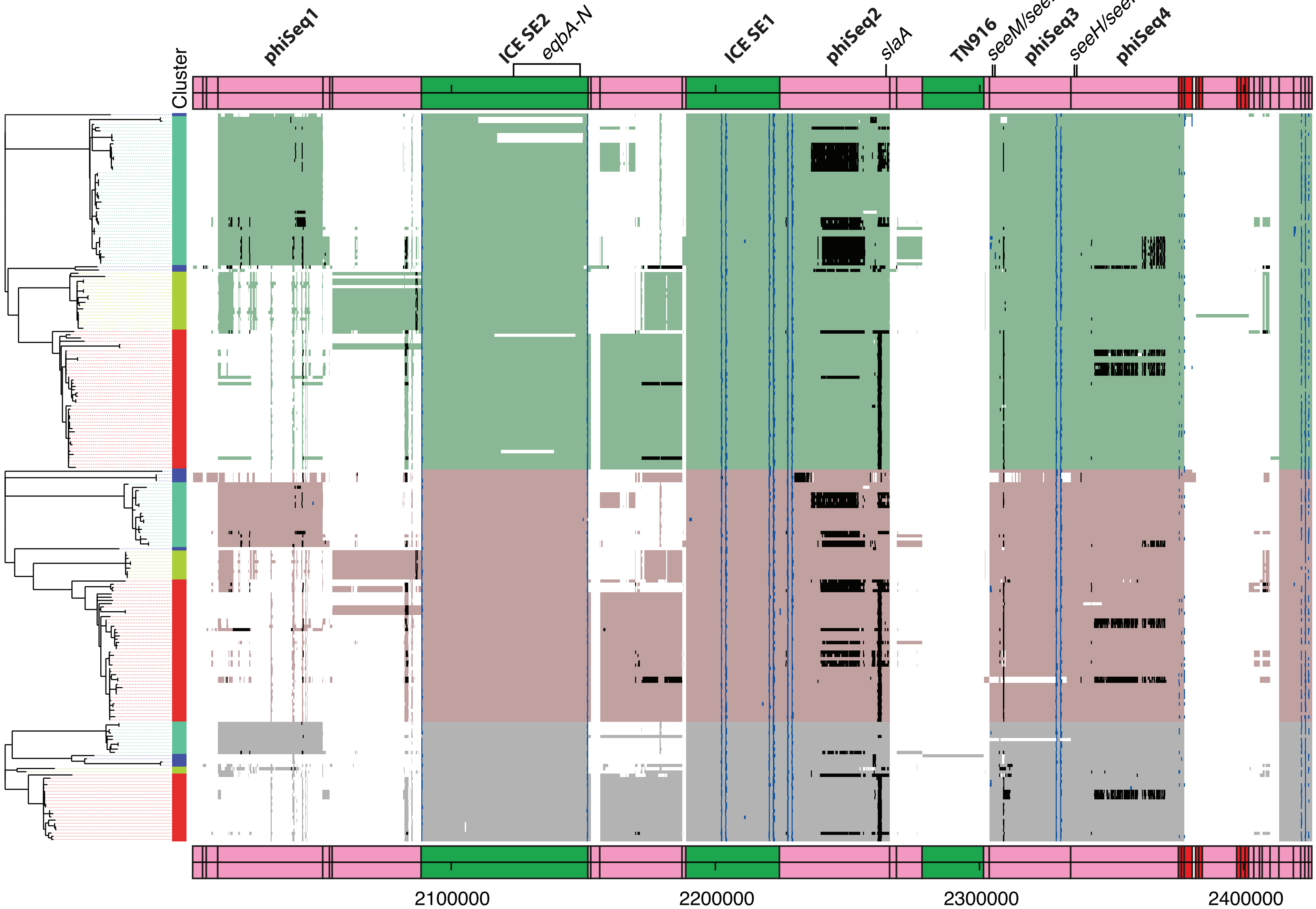

Supplementary Figure 10. Accessory genome insertions, duplications and IS elements in persistent, acute and other isolates. Three ML phylogenetic trees are shown in the left panel, created from only persistent isolates (top), only acute isolates (middle) and other isolates (bottom). For each, the column to the right of the tree indicates the BAPs cluster of the isolates in the species phylogeny in Fig. 1. In all cases, the topologies of the individual trees are consistent with the tree in Fig. 1. The right-hand panel shows coverage of the accessory genome in each isolate. To the top and bottom of the right panel are representations of the assembled accessory contigs from isolates in the study. The contig color gives an indication of the content of the contig. Pink = bacteriophage, green = integrative and conjugative element (ICE), red = IS element. Contigs present in the reference genome are labeled in bold above the panel, along with the location of some important virulence genes. Regions of single copy coverage are colored green in persistent isolates, red in acute isolates and gray in others. Regions of duplication are colored black, and deletions are white. IS element insertion locations are shown in blue.

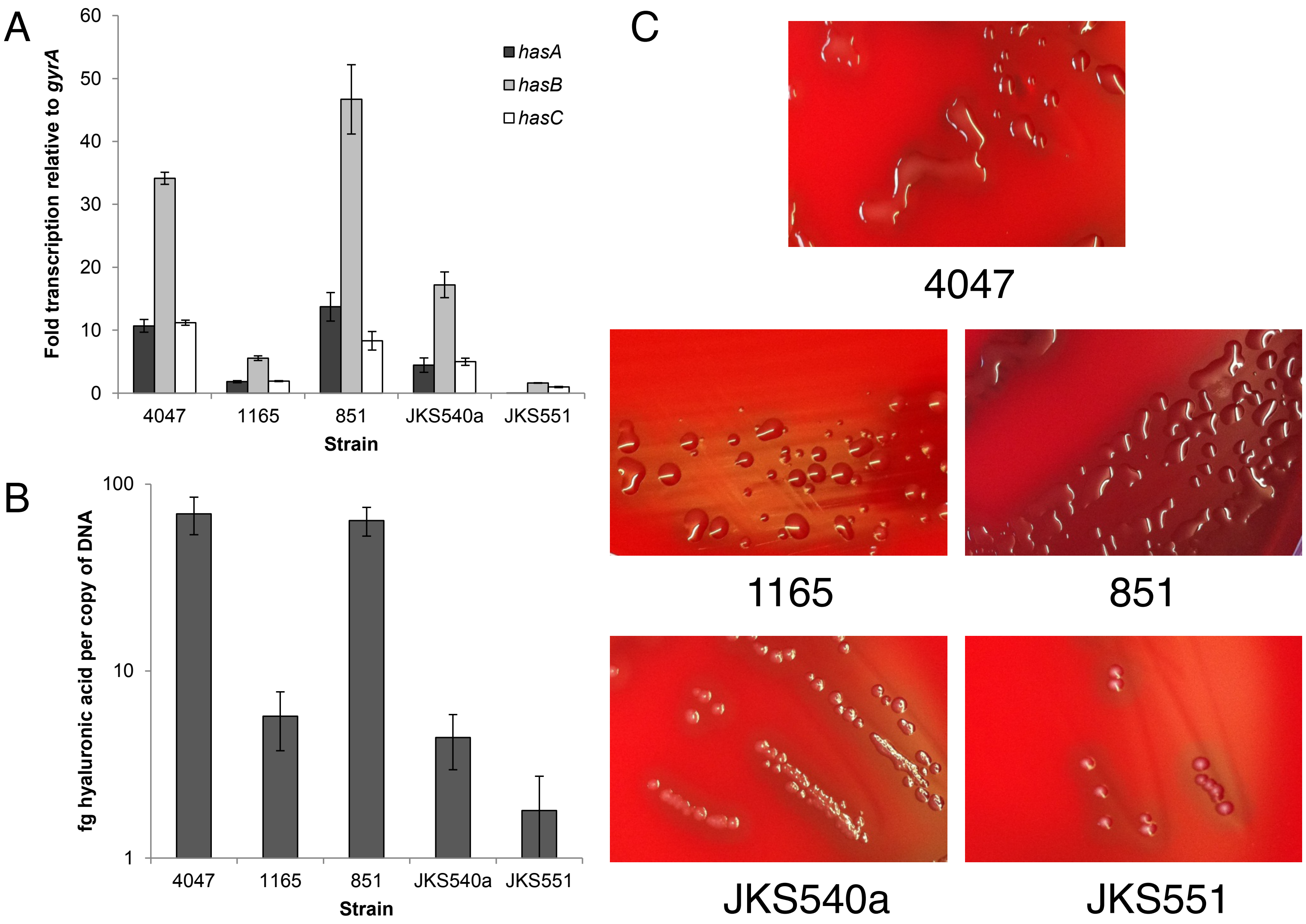

Supplementary Figure 11. Quantification of the effects of deletion and amplification of the *has* locus. Strain 1165 was isolated from a horse in Berwickshire (UK) on the 10th February 2005. Strain 851, which contains an amplified *has* locus, was isolated from the same horse as strain 1165, but on the 28th January 2005. Strain JKS540a was isolated from an outbreak of strangles in Essex on the 17th April 2007. Strain JKS551, which contains a deletion of *hasA* and part of *hasB* was recovered from a persistently affected horse at the same farm in Essex as JKS540a, but on the 18th July 2007. A) Graph showing the transcription of the *has* locus expressed relative to the *gyrA* housekeeping gene. Strain 4047 is the reference strain. Error bars indicate the 95% confidence intervals. B) Graph showing the amount of hyaluronic acid extracted from the surface of each strain relative to the amount of bacterial DNA present in each culture. Error bars indicate the 95% confidence intervals. C) Colony phenotypes of the strains grown overnight at 37 ^{o}C in an atmosphere containing 5 % CO_2 on COBA streptococcal selective agar.

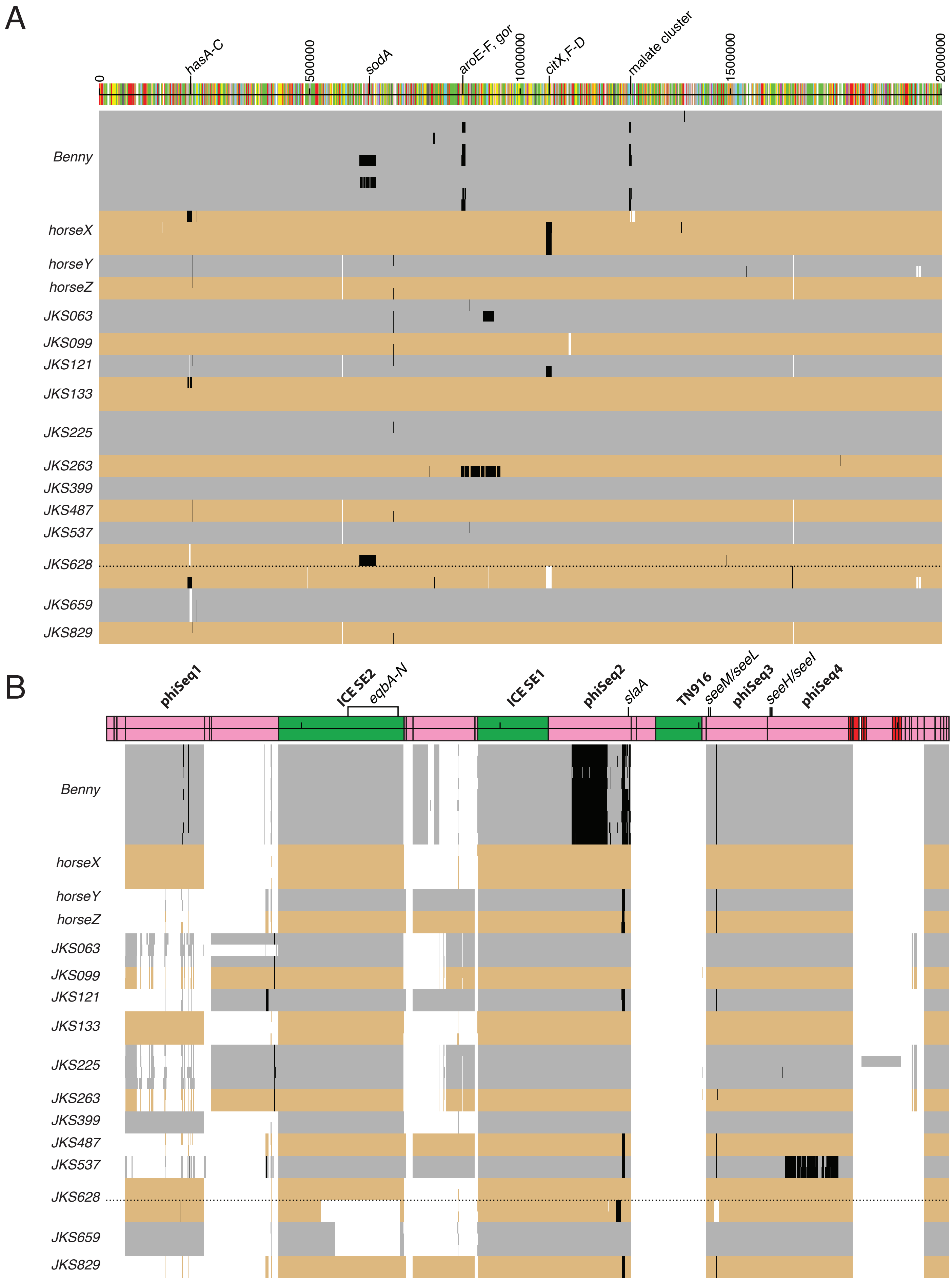

Supplementary Figure 12. Deletions and duplications of core (A) and accessory (B) regions of the genomes of carriage isolates from horses with more than one available sample. At the top of panel A is a representation of the annotation of the core genome, with relevant loci labelled. At the top of panel B are representations of the assembled accessory contigs from isolates in the study. The contig color gives an indication of the content of the contig. Pink = bacteriophage, green = integrative and conjugative element (ICE), red = IS element. Contigs present in the reference genome are labeled in bold above the panel, along with the location of some important virulence genes. In both panels, regions of duplication are colored black, and deletions are white with beige and grey indicating single coverage for isolates from alternating horses.
